# Measuring the value of accurate link prediction for network seeding

**DOI:** 10.1186/s40649-017-0037-3

**Published:** 2017-05-18

**Authors:** Yijin Wei, Gwen Spencer

**Affiliations:** 1 Center for Computational Engineering, MIT, Cambridge, MA USA; 2 Mathematics and Statistics, Smith College, Northampton, MA USA

**Keywords:** Influence maximization, Link prediction, Threshold spread, Network seeding, Optimization under uncertainty

## Abstract

**Merging two classic questions:**

The influence-maximization literature seeks small sets of individuals whose structural placement in the social network can drive large cascades of behavior. Optimization efforts to find the best *seed set* often assume perfect knowledge of the network topology. Unfortunately, social network links are rarely known in an exact way. When do seeding strategies based on less-than-accurate link prediction provide valuable insight?

**Our contribution:**

We introduce optimized-against-a-sample ($\text{OAS}$) performance to measure the value of optimizing seeding based on a noisy observation of a network. Our computational study investigates $\text{OAS}$ under several threshold-spread models in synthetic and real-world networks. Our focus is on measuring the value of imprecise link information. The level of investment in link prediction that is strategic appears to depend closely on spread model: in some parameter ranges investments in improving link prediction can pay substantial premiums in cascade size. For other ranges, such investments would be wasted. Several trends were remarkably consistent across topologies.

## Motivation and background

In the late 70s, Granovetter introduced the study of influence in social networks in the sociology literature [1]. In addition to ongoing inquiry in sociology, more recently this notion has been vigorously pursued in economics and computer science (Chen et al. [2] provide a thorough survey). For seminal contributions, also see [3–5], and Jackson’s popular textbook [6], as well as major contributions in the modernizing field of computational sociology [7, 8]. Planning variants focus on *maximizing influence* or *seeding behavior spread* by manipulating the initial behavior of a small number of key network members, known as *seeds* (see [4, 9]). Given an initial *seed set* of individuals, a spread model defines how each individual node will update its state in the next time step. These updates are usually based on the states of immediate neighbors, leading to *behavioral cascades* that spread through the network. Theoretical and computational studies have investigated a number of spread models including independent cascade, linear threshold [4], other threshold-based models [1], and complex contagion [8, 10]. Apparently similar spread models can lead to diverging implications about the form of highly influential sets of individuals: planners seek an *optimal seed set*.

Over the last decade, the capacity to collect large-scale network datasets has led to the emergence of modern network science. Some empirical observations have validated studied spread models, for example, Romero et al. observe a threshold-like complex contagion effect in spread of political hashtags on Twitter [11]. Further, implementation of seeding-style interventions is an increasingly accessible option for viral-marketing applications [12] at social-media companies like Facebook. As the field moves from theoretical insights about seeding towards implementation, increased attention has been directed towards practical considerations like scalable and distributed computation (moving beyond traditional asymptotic guarantees, e.g., [9, 13]) and concerns about whether underlying mathematical assumptions undermine the usefulness of known results.

For example, a ubiquitous assumption in the optimal seeding literature is that the planner has perfect knowledge of the network topology (as in [4, 9]), and that this topology is static. In practice, both of these assumptions seem quite unrealistic. Pointing out that the planner may be limited to local knowledge of network structure, Kim et al. explore an incomplete-information variant of the network seeding problem [14, 15]. Further, even if the planner has access to a global view of network structure, reliable observations of active network links for a past viral-marketing campaign may not translate reliably to the next product. Networks of interest may also be naturally dynamic (as discussed in [16–18]): social links are regularly formed and broken. Critiquing the assumption of precise knowledge of edge probabilities (which is essential to most provable approximation results under the Independent Cascade Model), He and Kempe introduce a model in which edge probabilities are selected from given intervals [19]. Very recent algorithmic studies of Chen et al. and He and Kempe build on this model, advocating for *robust influence maximization algorithms* [20, 21].

Indeed, link prediction is a cornerstone of modern network science. For example, see highly cited works like [22–24], and [17], and the useful recent survey of Lü and Zhou [25]. Given the myriad obstacles to obtaining perfectly accurate network topology, how does imperfect link prediction impact efforts to optimize network seeding? When do seeding strategies based on noisy observations of a social network yield valuable insight towards optimal seeding? Is imprecise link information more valuable in some settings than in others?

This paper focuses on two prominent spread models that are *time-indexed*: spread proceeds over a set of discrete time steps $t\in \{1,2,3,...\}$. At each time $t$ each node is either in state 0 or state 1. As these spread models build on disease transmission models from epidemiology, nodes with behavior 1 are often called *infected* (while behavior 0 nodes are *uninfected*).

*Irreversible uniform threshold spread (with infection threshold* $\tau$):

- Nodes in the *seed set* are infected for all time steps.
- For each node $v$ that is not in the seed set, at each time $t$: $v$ is infected at $t$ if and only if at least a $\tau$-fraction of $v$’s neighbors were infected at time $t-1$.

* Linear threshold spread from Kempe, Kleinberg, Tardos* [4]:

- For each node $v\in V(G)$, an infection threshold $\tau _v$ for node $v$ is realized uniformly-at-random from the interval [0, 1].
- Nodes in the *seed set* are infected for all time steps.
- For each node $v$ that is not in the seed set, at each time $t$: $v$ is infected at $t$ if and only if at least a $\tau _v$-fraction of $v$’s neighbors were infected at time $t-1$.

We are motivated to focus on uniform threshold spread both because of this model’s strong resemblance to Complex Contagion from sociology [8] (which has been qualitatively observed in real data [11]) and by the relative lack of theoretical traction for this model. Unlike more mathematically convenient models that have been widely studied (independent cascade, linear threshold), cascade size is not submodular under uniform threshold spread. Some promising algorithmic progress has been made for network-uncertainty variants of more mathematically convenient spread models [20, 21]. We observe that major differences emerge between uniform threshold spread and linear threshold spread (two models sometimes considered to be similar). Under the uniform threshold model, even varying the value of $\tau$ critically impacts the advantage of imperfect link prediction. This suggests that determining strategic levels of investment in reducing link-prediction error may require close study of the operating spread mechanism. As noted in He and Kempe [21], a wide range of network and spread-model features may be varied experimentally (and may be significant in determining outcomes): our study is necessarily limited.

In this paper, we pose and explore a set of questions that we hope will motivate further study for a range of spread models and topologies.

### Our contribution

We conduct a computational study to explore how imperfect link prediction affects the performance of “optimized”? (or near-optimized) seeding strategies. To formalize this notion, we introduce optimized-against-a-sample ($\text{OAS}$) Performance. Given a noisy sample observation $G'$ of an original network $G$, some seed set $V'$ is optimal *with respect to the noisy network*, $G'$. In turn, this seed set $V'$ has some performance *in the original network*, $G$. We define $\text{OAS}$ performance as the expectation of $V'$s performance in $G$ (with respect to some distribution over noisy samples $G'$).

Focusing on Uniform Threshold spread and Linear Threshold spread, we investigate how $\text{OAS}$ Performance compares to two practical reference points. First, we compare $\text{OAS}$ Performance to the performance achievable by a planner who is completely ignorant of network structure (and must effectively choose a seed set at random). Our goal is to provide such a planner with a message of the flavor, “Investments in gathering link information of a certain quality will allow your optimized seeding strategies to reliably outperform your current no-information strategy.” Second, we compare $\text{OAS}$ Performance to the performance achievable by a planner with perfect knowledge of network structure. Here, we hope to advise a planner who already has access to good link-prediction methods: “How large a margin can gained by further investments in improving link prediction?” Both reference points are important to understanding strategic levels of investment in link-prediction capability.

Critically, $\text{OAS}$ should not be viewed as an optimization algorithm: it is a measurement to describe how valuable imperfect network-structure information is towards planning seeding. Network seeding under many spread models of interest gives NP-hard problems: a planner with perfect link information does not escape from this challenge. The experiments in this paper consider a planner who applies *traditional* and *modified greedy* seed-selection methods^1^ to approximate $V'$, but similar studies with respect to alternative seed-selection algorithms would also be of great interest. We make $\text{OAS}$ measurements in synthetic and real network datasets (small world, scale-free, email-exchange, and messenger-app contacts). To measure behavior over a range of threshold values and provide confidence intervals, for each network we consider 80,000+ realizations of $G'$.

Surprisingly, we find that higher Uniform Threshold values increase how much link-prediction error is *tolerable* in planning complete cascades. We say that a rate of link-prediction error is *tolerable* if $V'$ remains competitive with seeding based on perfectly accurate link information, and most realizations of $G'$ yield a $V'$ with performance that exceeds random seeding. We also observe a second style of *tolerance* against link-prediction error when $\text{OAS}$ performance remains substantially above the performance of random seeding despite remarkably high link prediction error.

For $\text{OAS}$ based on both *traditional* and *modified greedy* seeding, highly accurate link prediction appears essential when thresholds are very low (both in synthetic and real network datasets). In contrast, at higher thresholds, $\text{OAS}$ reliably yields significant insight in optimizing seeding, even for high rates of link-prediction error. For $\text{OAS}$ where an estimate of $V'$ is found with *modified greedy seeding*, we observe that in planning full cascades, the stability of (near-) optimized seeding strategies (against noise in link prediction) increases with node thresholds. *For high thresholds, a seed set that will truly “go viral” can be found by modified greedy seeding even from a quite-noisy view of the network structure.* At lower budgets, where infections spread modestly but do not “go viral,” damage to seeding performance due to noisy link prediction appears immediate: we observe no stability effect. If instead, $V'$ is estimated with *traditional greedy seeding*, for high thresholds in scale-free networks we observe a modest but remarkably stable $\text{OAS}$ advantage even at the highest levels of link error. *For high thresholds in scale-free networks, even a highly noisy view of the network can steer traditional greedy seeding to choose a modestly effective seed set.*

Finally, under the Linear Threshold Model, even when subject to surprisingly high levels of link-prediction error, $\text{OAS}$ can still provide substantial reliable insight towards seeding. Across a range of budgets for seeding, we find that the behavior of $\text{OAS}$ in a smaller synthetic scale-free network anticipates the behavior we observe in two larger real network examples. Significant stability of (near-) optimized seeding strategies, despite intensely noisy link information, is observed across a range of budgets for the Linear Threshold Model. Throughout, we comment on similarities and contrasts between $\text{OAS}$ measurements that emerge from the two greedy-seeding mechanisms we consider for approximating $V'$ in $G'$.

## Methods

Suppose we are given an *original network* $G=(V(G), E(G))$, a spread model *S*, and some probability distribution *P* over noisy observations of the edge set of the *original network*, $E(G)$. Uncertainty is limited to link prediction: assume all observations from *P* have node set $V(G)$.

### Generating a noisy observation of $G$

Let $G'$ denote a noisy observation of the *original network* realized from distribution *P*. Many different distributions *P* over observed links may be plausible and justifiable based on the research literature in link prediction. We adopt a simple model for *P* based on independent *false negative* events and *false positive* events for link prediction:

*False negative rate* ($p_{\text {neg}}$) For each $e\in E(G)$, then $e\in E(G')$ with probability $1-p_{\text {neg}}$.

*False positive rate* ($p_{\text {pos}}$) For each $e\notin E(G)$, then $e\in E(G')$ with probability $p_{\text {pos}}$.

This is similar to the uncertainty model used by Adiga et al. in their algorithmic study of the Independent Cascade Model [26].

While $p_{\text {neg}}$ and $p_{\text {pos}}$ could be varied separately, our initial exploration will assume that $E_P[|E(G')|]\approx |E(G)|$, so that the density of $G$ is roughly maintained in samples from *P* (in the expected value sense).^2^ To force this, equate the expected number of edges that exist but are not observed, and the expected number of edges that do not exist but are observed:

1 $$\begin{aligned} p_{\text {neg}}|E(G)|=\Bigg [{|V(G)|\atopwithdelims ()2}-|E(G)|\Bigg ]p_{\text {pos}}. \end{aligned}$$

Then we obtain

2 $$\begin{aligned} p_{\text {pos}}=\frac{p_{\text {neg}}|E(G)|}{{|V(G)|\atopwithdelims ()2}-|E(G)|}. \end{aligned}$$

A consequence of this definition of $p_{\text {pos}}$ in sparse graphs is that even high $p_{\text {neg}}$ can yield an observed graph $G'$ that has a higher likelihood of having an edge where $G$ contained an edge than where $G$ had no edge. In other words, the noisy observation resulting from high $p_{\text {neg}}$ still retains some information about the original network.

### Determining budget $b$ for seeding.

**Fig. 1:**
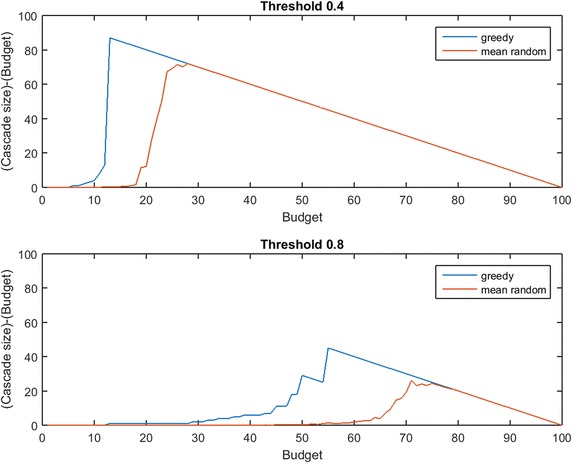
Budgets where strategic seeding is relevant. When the budget for seeding, $b$, is very low or very high, randomly chosen seed sets are competitive with strategically chosen seed sets. In the *top panel*, for node threshold $\tau =0.4$, greedy seeding (shown in *blue*) outperforms random selection for $b\in [5,27]$. Above $b=28$ both methods give complete cascades (additional spending on seeding is wasted). In the *bottom panel*, for node threshold $\tau =0.8$, greedy seeding outperforms random selection for $b\in [12,75]$, but the additive advantage of greedy selection for $b\le 28$ is extremely small

The budget for seeding, $b$, limits how many initial nodes may be infected at time 0 by the planner. When budgets are very high or very low, the additive difference in cascade size between a strategically chosen seed set and a randomly selected seed set is small. Figure 1 illustrates this point by comparing greedy seed selection (assuming perfect link information) and mean random-seeding performance across all possible budgets in a 100-node network.

Figure 1 informs our experimental design: at budgets where perfect link prediction yields no advantage over random seeding, imperfect link prediction cannot possibly provide value to the planner. Any meaningful measurement of the value of imperfect link prediction must be conducted at a budget, $b$, where *a very good seed set exists, but where the chance of randomly guessing a good seed set is low*. Budget levels that are meaningful will vary strongly depending on node threshold $\tau$ (as shown in Fig. 1), and will also depend on the structure of $G$.

Our first set of experiments aims to compare measurements across networks and threshold levels: we must propose a systematic way of selecting a meaningful budget, $b$. For fixed $G$ and spread model, we begin by choosing the smallest $b$ so that at least $98\%$+ of the planner’s greedy attempts to seed $G'$ result in full cascades for $G'$. This initial choice ensures that poor performance of $V'$ in $G$ is due to the structural differences between $G'$ and $G$, and not to $V's$ suboptimality in $G'$.^3^ Practically speaking, our planner designs a seed set they believe (based on $G'$) will cause a full cascade, then observes some actual impact of their seed set in $G$. Budgets used in all experiments are listed in the corresponding figures.

Our initial experiments expose that budgets planned based on $G'$ in networks with heavily skewed degree distributions (as in many real-data examples) can lead to wasteful levels of seeding. Thus, in considering real-data examples we seed at a budget sufficient for greedy seeding with perfect information to cause a complete cascade in $G$, but not necessarily in $G'$. This new $b$ corresponds to the blue peaks in Fig. 1. At the end of our study of Uniform Thresholds, we also probe $\text{OAS}$ at a fraction of this level (to the left of the blue peaks in Fig. 1). In studying the Linear Threshold Model, we also consider a range of budgets that give partial cascades.

### Optimizing seeding for a noisy observation of *E*(*G*)

Since network seeding under many spread models of interest gives NP-hard problems, the planner cannot optimize exactly in $G'$. In this paper, we consider a planner who adopts a greedy approach to seed selection. We will describe experiments both for a *traditional greedy* algorithm and a *modified greedy* algorithm.

The *traditional greedy* algorithm sequentially selects a set of seed nodes, $S$. Starting from $S=\emptyset$, until the budget is reached, the node that gives the highest marginal increase in cascade size (beyond the cascade size caused by the current *S*) is added to *S*. When no node provides an increase in cascade size, the next seed is chosen at random. To reflect that the planner’s estimate of $V'$ is chosen in this *traditional greedy* way, we henceforth refer to $\text{OAS}_{\text {tg}}$. Computing cascade-size margins for each candidate seed becomes slow for large networks (particularly when the experiment is replicated many times at each value of $p_{\text {neg}}$ across the range [0, 1]). For example, in a 1000 node network, allocating 100 seeds in $G'$ requires roughly 100,000 simulations of the spread process across a 1000 node graph. Since $G'$ is randomly realized, to have a sense of “typical behavior,” this process must be replicated several times at each $p_{\text {neg}}$ value of interest.

The *modified greedy* algorithm prioritizes seed choices that maximize progress towards meeting the thresholds of (many) neighbors. Precisely, let $X$ denote the seed set already chosen by the planner, and $\delta (v)$ denote the degree of node $v$ in $G'$. For each $v\in V(G')$, let $\delta _X(v)$ denote the number of neighbors of $v$ in $X$. Finally, let $\delta _{\hat{X}}(v)$ denote $\lceil \tau _v*|\delta (v)|\rceil -|\delta _X(v)|$, the number of additional seeds required in $v'$s neighborhood for $v'$s threshold to be met. Then, the next node selected by the planner to add to $X$ will be the non-seed node that maximizes

$$\begin{aligned} g(v)=\sum _{\{i\in \delta (v): \delta _{\hat{X}}(i)>0\}} \Bigg (\frac{1}{\delta _{\hat{X}}(i)}\Bigg ). \end{aligned}$$

To reflect that the planner’s estimate of $V'$ is chosen in this *modified greedy* way, we henceforth refer to $\text {OAS}_{\text {mg}}$.

Our entire suite of experiments could be replicated to study the value of link prediction for planners who employ some alternative seed-selection method (greedy or otherwise).

## Experimental results

We investigate empirical $\text {OAS}_{\text {mg}}$ and $\text {OAS}_{\text {tg}}$ measurements in several classes of synthetic graphs (small-world, scale-free) as well as real network data (Facebook-like messenger app at University of California, Irvine and a Spanish university email-exchange network). To measure $\text{OAS}$ behavior over a range of infection thresholds and explore the distribution of $V'$s performance in $G$, for each network described below we conduct 80,000+ realizations of $G'$. A summary of network statistics is shown in Table 1.

**Table 1:** Summary of network statistics

	Number of nodes	Number of edges	Average degree
Small-world network (comm. size: 10, $p=0.4$)	300	1854	12.4
Small-world network (comm. size: 10, $p=0.6$)	300	1697	11.3
Small-world network (comm. size: 20, $p=0.4$)	300	3135	20.9
Scale-free network (init. society of 40)	300	2484	16.5
Scale-free network (init. society of 120)	300	2481	16.5
UCI messenger-app network	1281	13,010	20.3
Spanish email-exchange network	1133	5451	9.6

In the following figures, the mean performance of a randomly selected $b$-node seed set is plotted in red. This represents the typical performance of a seeding strategy that uses *no information* about the topology of $G$. We find that this mean random performance sometimes infects very few nodes beyond the seeding budget $b$ (plotted in yellow), despite the fact that $b$ is sufficient to cause a complete cascade in both $G$ and $G'$ (in this section). This random mean provides a minimal baseline: any strategy that does not allow a planner to consistently exceed a random guess has little value. When is greedy seeding that relies on noisy information about *G*’s topology *reliably better* than a typical random guess (that uses no information about *G*’s topology)?

First we report all results for the Irreversible Uniform Threshold Spread Model, then we describe results for the Linear Threshold Model.

### Synthetic networks

#### Small-world networks

We generate three small-world networks on 300 nodes by following the random rewiring procedure of Watts and Strogatz [27]. We start this rewiring procedure from a network that consists of small communities of normally distributed sizes. Initially, each node is connected to every node in the same community by an edge (and is connected to no other nodes). With probability *p*, each edge is rewired to a node chosen uniformly at random outside its community, with duplicate edges forbidden; otherwise we retain the original edge. Three small-world networks on 300 nodes are generated for varying combinations of initial mean community size and rewiring probability *p*, as listed in Table 1.

**Fig. 2:**
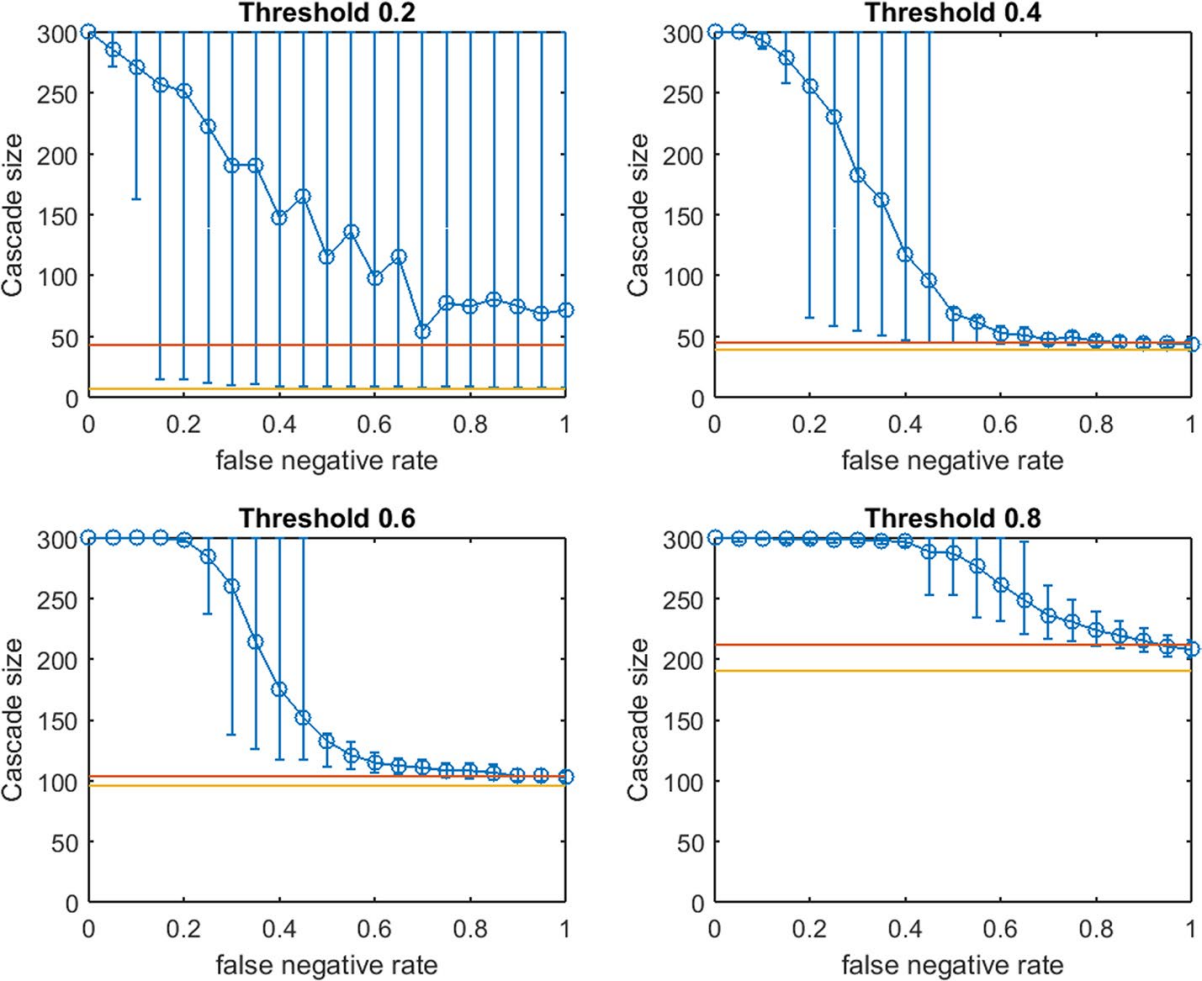
Increasing link-prediction error in a small-world network ($\text {OAS}_{\text {mg}}$). $\text {OAS}_{\text {mg}}$ as a function of false negative rate in a small-world network (rewiring probability of 0.4, mean community size of 10, standard deviation 5). Nodes have uniform infection thresholds of 0.2 (*upper left*), 0.4 (*upper right*), 0.6 (*lower left*), 0.8 (*lower right*). Larger link-prediction error is modeled by larger false negative rate.* Horizontal lines* plot seeding budget $b$ (in *yellow*) mean performance of a randomly selected seed set (in *red*). *Modified greedy* allocation of $b$ seeds causes a complete cascade in $G'$ (300 nodes). Budgets are (7, 39, 96, 191)

**Fig. 3:**
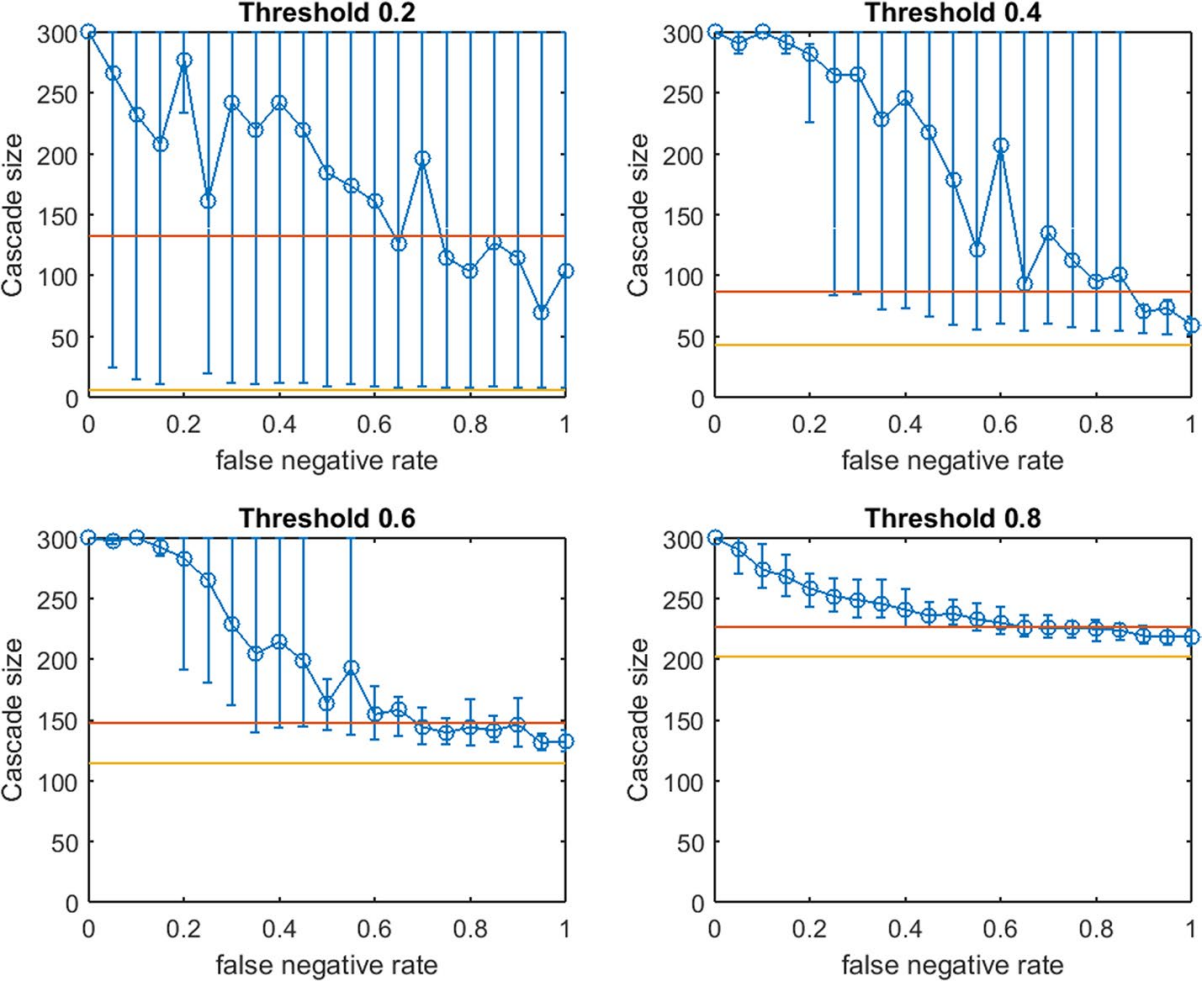
Increasing link-prediction error in a small-world network ($\text {OAS}_{\text {tg}}$). The experiment from Fig. 2 is replicated with the *traditional greedy* algorithm. $\text {OAS}_{\text {tg}}$ is depicted as a function of false negative rate in a small-world network. *Traditional greedy* allocation of $b$ seeds causes a complete cascade in $G'$ (300 nodes). Budgets are (9, 43, 114, 202). Since budgets required by *traditional greedy* are higher than those required by *modified greedy*, the average performance of a random seed set in $G$ (shown in *red*) is higher here than for each corresponding subplot in Fig. 2

Figure 2 depicts empirical $\text {OAS}_{\text {mg}}$ in a small-world network (mean community size 10 with standard deviation 5, $p=0.4$) at increasing false negative rates for link prediction. Performance distributions are highly asymmetric so standard confidence intervals are not appropriate: 10th–90th percentile observations are displayed (based on $V'$ from 100 samples of $G'$). Each panel is labeled by the uniform infection threshold, $\tau$. Budgets, $b$, are shown in yellow. The mean performance of random seed selection is shown in red. Figure 3 replicates the same experiment for $\text {OAS}_{\text {tg}}$. Since the *traditional greedy* algorithm is slower than *modified greedy*, as shown in Fig. 3 means and percentile intervals at each $p_{\text {neg}}$ are computed based on 25 samples of $G'$.

**Fig. 4:**
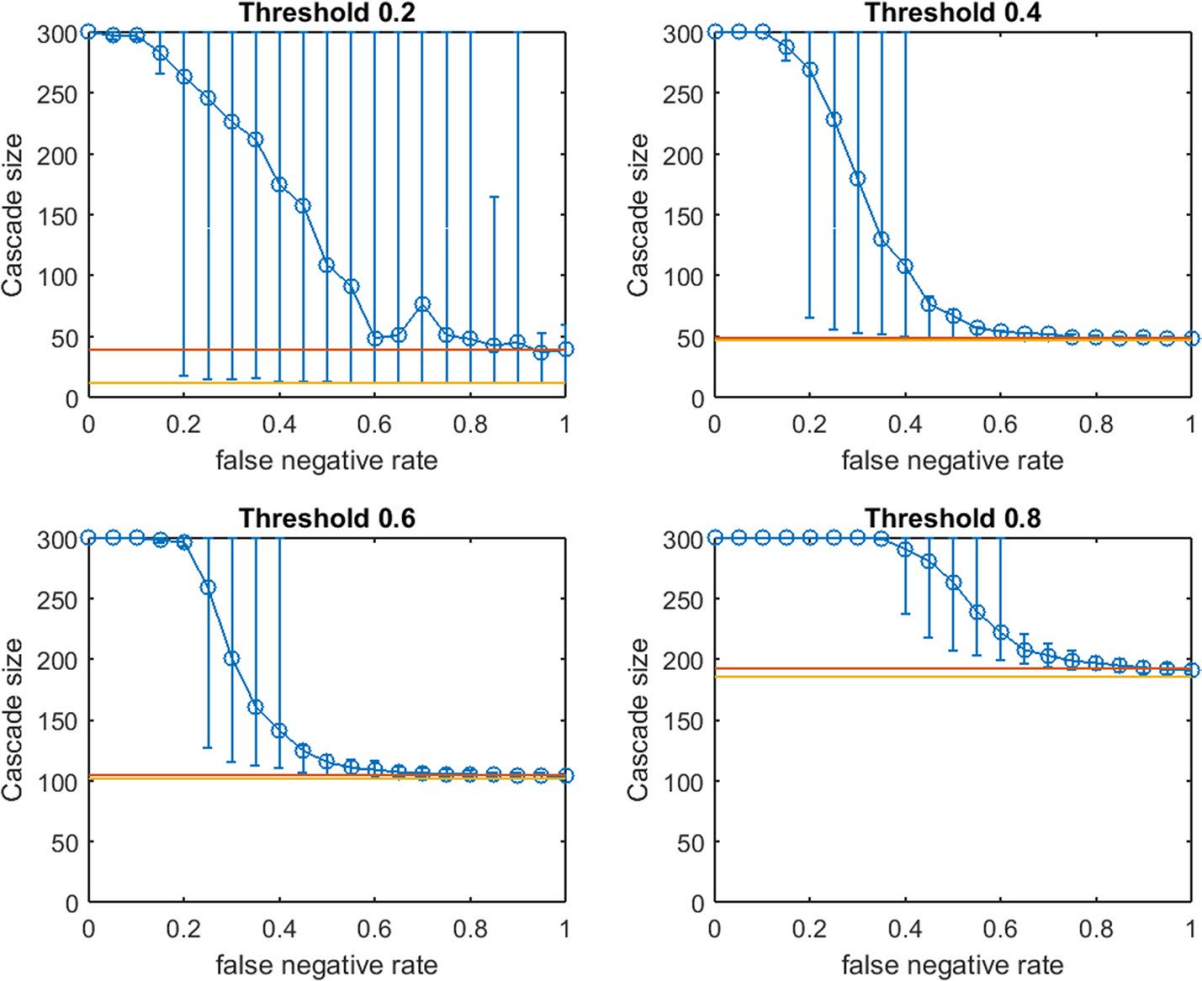
Increasing link-prediction error in a small-world network (larger communities, $\text {OAS}_{\text {mg}}$). $\text {OAS}_{\text {mg}}$ as a function of false negative rate in a small-world network (rewiring probability of 0.4, mean community size of 20, standard deviation 5). Nodes have uniform infection thresholds of 0.2 (*upper left*), 0.4 (*upper right*), 0.6 (*lower left*), 0.8 (*lower right*). Larger link-prediction error is modeled by larger false negative rate.* Horizontal lines* plot seeding budget $b$ and mean performance of a randomly selected seed set. *Modified greedy* allocation of $b$ seeds causes a complete cascade in $G'$ (300 nodes). Budgets are (12, 47, 102, 186)

First we note commonalities of Figs. 2 and 3. For all infection thresholds, when $p_{\text {neg}}$ is very small, greedy seeding with respect to the noisy sample $G'$ reliably outperforms random seeding. As $p_{\text {neg}}$ increases, $\text{OAS}$ performance passes through a region of steep decrease with broad distribution of observed cascade sizes ($V'$ has widely varying performance in $G$). As $p_{\text {neg}}$ becomes large, optimizing-against- a-sample appears to provide little advantage over random seed selection. This trend is intuitive: optimizing seeding with respect to noisier network observations yields progressively worse performance in the original network.

In Fig.  2, for infection threshold $\tau =0.4$, $p_{\text {neg}}=0.45$ is the lowest false negative rate for which the 10th–90th-percentile interval for $V'$s performance contains the mean random-seeding performance (shown in red). That is, when the false negative rate for link prediction surpasses 0.45, optimizing seeding with respect to a noisy observation of the network may frequently perform no better than a randomly selected seed set. For lower false negative rates, however, optimizing-against-a-sample appears to provide a substantial and reliable advantage over random seed selection. We note that the false negative rate at which the 10th–90th-percentile interval first includes the mean random seeding performance seems to increase at larger infection thresholds. A similar observation holds for $\text {OAS}_{\text {tg}}$ with *traditional greedy* seeding in Fig. 3. Doubling the mean size of the initial communities to 20 (with standard deviation 5, rewiring $p=0.4$), we observe very similar behavior (see Fig. 4 for $\text {OAS}_{\text {mg}}$).

For the *modified greedy* algorithm, Figs. 2 and 4 show that at higher infection thresholds $\text {OAS}_{\text {mg}}$ seems to match the performance of greedy selection with perfect link information (300 nodes) for longer initial intervals of $p_{\text {neg}}$ values. Remarkably, as shown in Fig. 2: for $\tau =0.8$, up to $p_{\text {neg}}=0.4$, greedy seeding in the noisy sample network $G'$ consistently achieves a practically complete cascade in the true network, $G$. Even quite-noisy link information about $G$ allows the *modified greedy* planner to consistently perform extremely well.^4^ As thresholds increase, it appears that precise link information is less and less important in remaining competitive with seeding based on perfect link information.

At the highest threshold of 0.8, we note a strong contrast between $\text{OAS}$ based on *modified greedy* vs. *traditional greedy* seeding (Figs. 2 vs. 3). In Fig. 3, as threshold increases, the range of $p_{\text {neg}}$ where $\text {OAS}_{\text {tg}}$ is competitive with perfect-information seeding initially appears to be expanding (as in Figs. 2, 4 for *modified greedy*). Then, at threshold 0.8, the shape of the $\text {OAS}_{\text {tg}}$ curve changes: as $p_{\text {neg}}$ increases, $\text {OAS}_{\text {tg}}$ immediately begins to decline. Note that Figs.  2 and 3 refer to *the same* small world network. For the highest threshold of 0.8, $\text {OAS}_{\text {mg}}$ subject to significant link-prediction error of $p_{\text {neg}}=0.4$ reliably delivers a complete cascade. At the same level of link-prediction error, *and despite a higher budget*, $\text {OAS}_{\text {tg}}$ barely outperforms random-seeding performance. We hypothesize that at high thresholds, the *traditional greedy* algorithm is susceptible to “over-fitting” to the observed edges, $E(G')$. Seeds are chosen to maximize cascade margins in $G'$ that frequently are not realized in $G$. For example, the discrepancy between $E(G')$ and *E*(*G*) leads to some node threshold values, $\lceil \tau *\delta (v)\rceil$, being higher than the planner expected from observing $G'$. Interestingly, damage due to such “over-fitting” is not apparent at lower thresholds (for $\tau$ of 0.2, 0.4, and 0.6, Figs. 2, 3 are similar), but this damage becomes very obvious at the highest threshold (0.8).

**Fig. 5:**
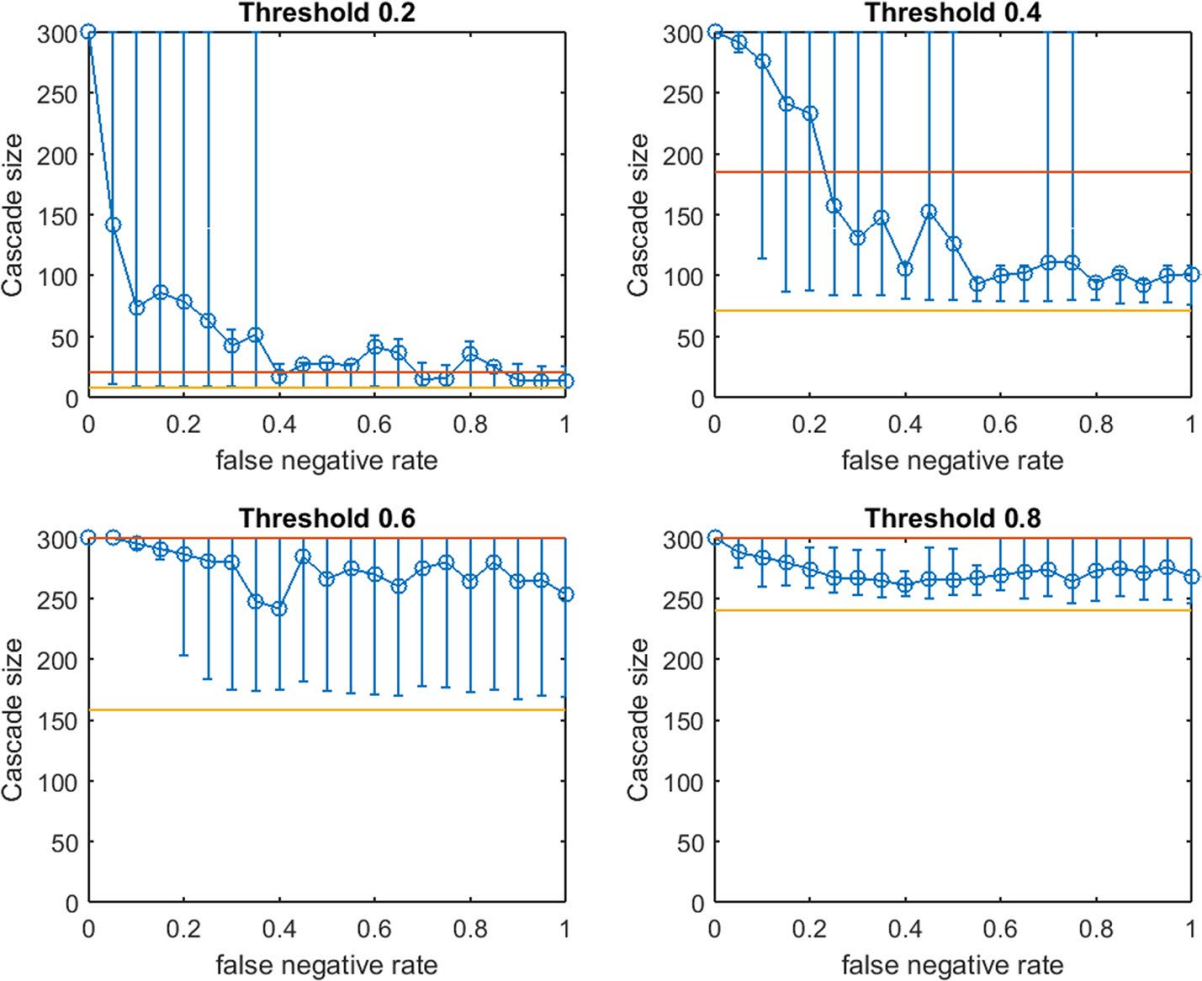
Increasing link-prediction error in a small-world network (larger communities, $\text {OAS}_{\text {tg}}$). The experiment from Fig. 4 is replicated with the *traditional greedy* algorithm. $\text {OAS}_{\text {tg}}$ as a function of false negative rate in a small-world network (rewiring probability of 0.4, mean community size of 20, standard deviation 5). *Traditional greedy* allocation of $b$ seeds causes a complete cascade in $G'$ (300 nodes). Budgets are (10, 68, 153, 235). At all but the lowest threshold, budgets required by *traditional greedy* in $G'$ to achieve full cascades are much higher than those required by *modified greedy*: the average performance of a random seed set in $G$ (shown in *red*) is higher for each subplot than in Fig. 4

A further weakness of applying *traditional greedy* seeding based on $G'$ is exposed in Fig. 5. Figure 5 replicates our $\text {OAS}_{\text {mg}}$ larger-communities experiment from Fig. 4 but with $\text {OAS}_{\text {tg}}$. Our experimental budget-selection criteria until now is that $b$ should allow the planner to achieve a full cascade in $G'$ for $98\%+$ of samples for $G'$. Because *traditional greedy* is so inefficient in seeding highly clustered networks with high thresholds, as shown in Fig. 5 the budgets chosen for larger fractional thresholds are much larger than under *modified greedy* seeding (see contrast with Fig. 4 for *the same* small-world network). In fact, *traditional greedy* seeding can be so wasteful for higher thresholds that the resulting budgets allow randomly selected seed sets (shown in red) to consistently deliver complete cascades in $G$—even as the planner’s efforts based on *traditional greedy* seeding in $G'$ usually deliver only partial cascades.^5^ In Fig. 5 we observe that at higher thresholds (above $p_{\text {neg}}=0.5$ for $\tau =0.4$, and across $p_{\text {neg}}$ for $\tau \ge 0.6$), *traditional greedy* seeding in a noisy network “over-fits” to such an extent as to *significantly damage the planner*: $\text {OAS}_{\text {tg}}$ is actually *reliably worse* than random seeding performance (shown in red).

While the contrast between Figs. 2 and 3 shows that in small-world networks $\text {OAS}_{\text {tg}}$ may be particularly susceptible to *over-fitting at higher uniform thresholds*, when considering larger community sizes, the contrast between Figs. 4 and 5 shows that both significant *over-fitting* and *overspending* may impact a *traditional greedy* planner with access only to noisy $G'$ (except at the lowest thresholds).

#### Scale-free networks

Networks with power-law degree distributions are often called *scale-free networks*. Our test scale-free network on 300 nodes is generated with preferential attachment [28]. We start with an initial base community of 40 nodes with average-degree 16 (binomial degree distribution). Next, 260 new nodes are added gradually to the network. Each new node makes eight attempts to connect to existing nodes. The probability that an edge exists between the newly added node *v* and an arbitrary existing node *i* follows the linear preferential-attachment function [29].

3 $$\begin{aligned} Pr[(v, i)] = \deg (i)/\Sigma _j \deg (j). \end{aligned}$$

**Fig. 6:**
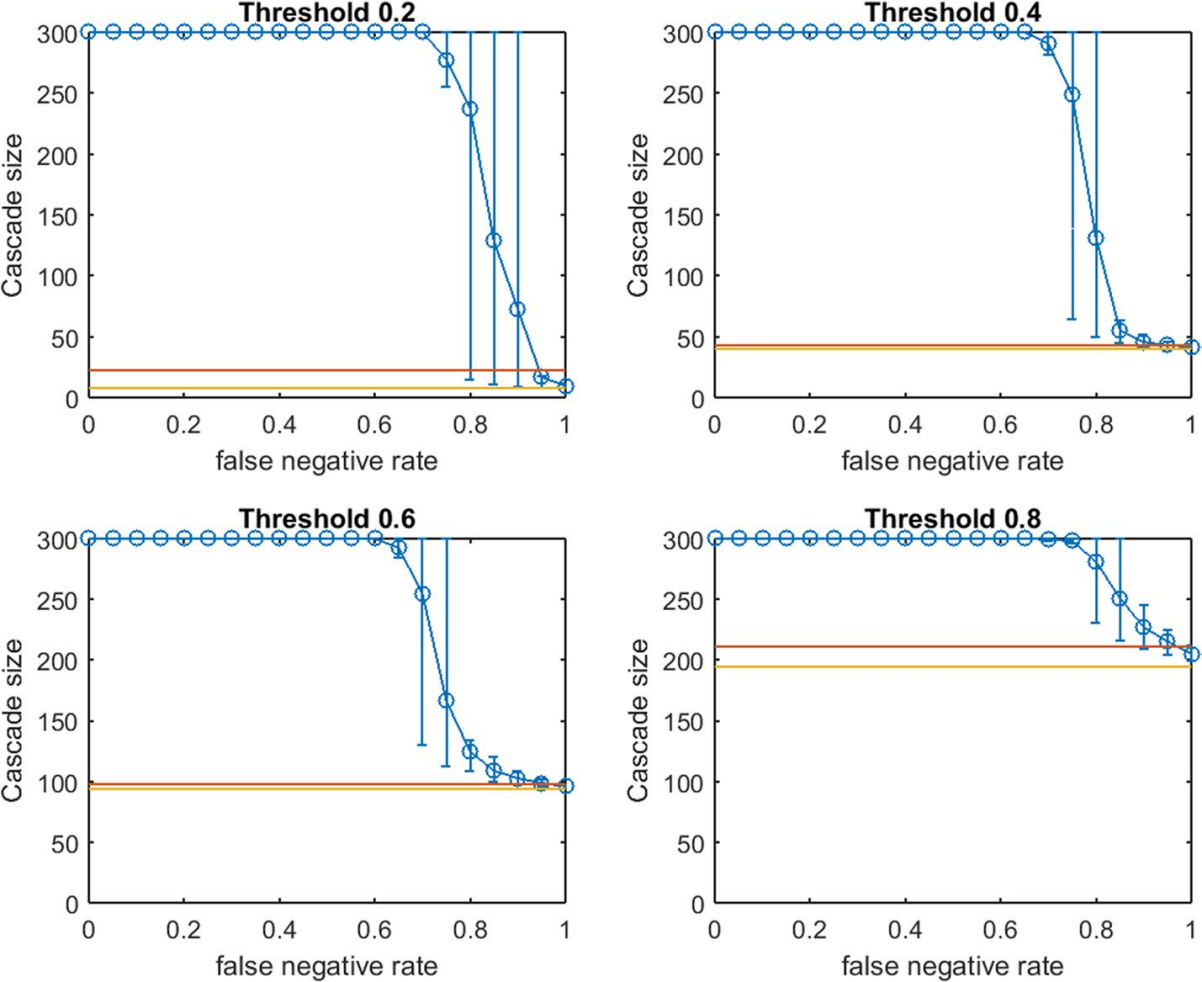
Increasing link-prediction error in a scale-free network ($\text {OAS}_{\text {mg}}$). $\text {OAS}_{\text {mg}}$ as a function of false negative rate in a scale-free network (initial society of 40 followed by preferential attachment of 260 nodes). Nodes have uniform infection thresholds 0.2, 0.4, 0.6, 0.8. Larger link prediction error rate is modeled through larger false negative rate. *Modified greedy* allocation of $b$ seeds causes a complete cascade in $G'$ (300 nodes). Budgets are (8, 40, 94, 194)

While preferential attachment builds a network structure quite different from the small-world network, there are qualitative similarities between previous figures and Fig. 6. Again, at smaller $p_{\text {neg}}$, $\text {OAS}_{\text {mg}}$ matches perfect-information performance. Again, we observe a steep decline in $\text {OAS}_{\text {mg}}$ with a broad distribution until $\text {OAS}_{\text {mg}}$ is roughly equal to mean random seed selection. This decline is now concentrated at higher $p_{\text {neg}}$ for all infection thresholds. Again the 10th–90th percentile interval first contains random mean performance at a $p_{\text {neg}}$ value that appears to (slightly) increase with node threshold $\tau$.

In contrast with figures for small-world networks, Fig. 6 has very long intervals of $p_{\text {neg}}$ values where $\text {OAS}_{\text {mg}}$ causes a full cascade. This is intuitive: since $G$ is a preferential-attachment network, the optimal seed set for $G$ will contain a small number of the highest degree nodes (many nodes in a preferential-attachment network see mostly such neighbors). Higher values of false negative rate, $p_{\text {neg}}$, “flatten-out” the degree distribution of $G$ (at $p_{\text {neg}}=0.5$ the maximum degree of $G'$ is roughly half the maximum degree of $G$). As a result, the budget required to cause a complete cascade in $G'$ is more than sufficient to cause a complete cascade in $G$: thus complete cascades are achieved by $\text {OAS}_{\text {mg}}$ until the structural differences between $G$ and $G'$ are extreme.

**Fig. 7:**
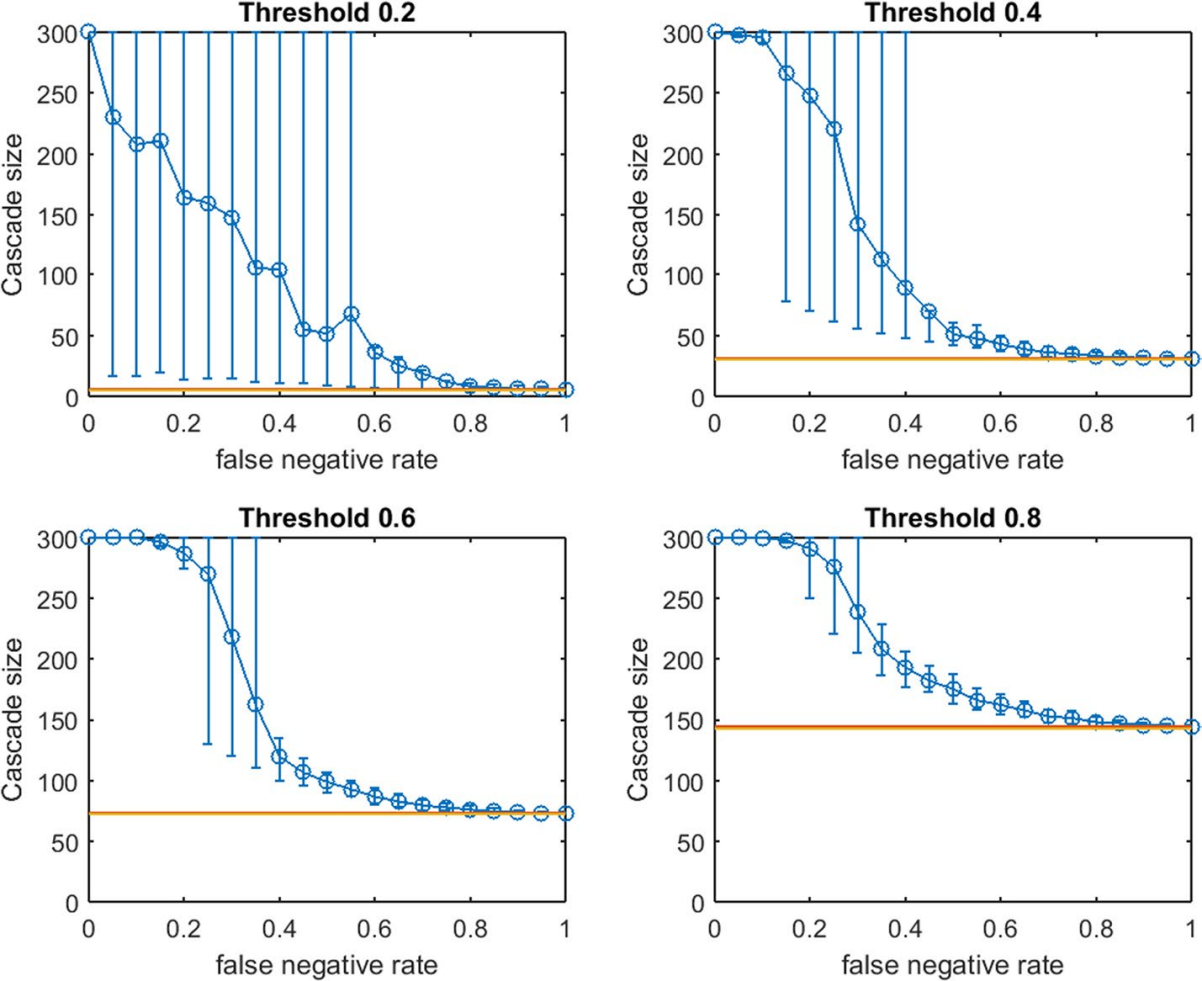
Increasing link-prediction error in a scale-free network (smaller budget, $\text {OAS}_{\text {mg}}$). $\text {OAS}_{\text {mg}}$ as a function of false negative rate in a scale-free network (initial society of 40 followed by preferential attachment of 260 nodes). Nodes have uniform infection thresholds 0.2, 0.4, 0.6, 0.8. This figure replicates the experiments from Fig. 6 for a smaller budget (sufficient for *modified greedy* to give a complete cascade in $G$). Budgets are (5, 30, 72, 143)

To check this understanding, we consider seeding our scale-free network at a smaller budget: we let $b$ be the lowest budget sufficient to cause a full cascade in $G$ under greedy seeding. Thus we obtain Fig. 7. At these lower budgets we obtain results that are qualitatively very similar to our observations in small-world networks (Figs. 2, 3, 4). Budgets are now so small that random seeding can completely fail to cause new infections (the red horizontal line depicting random seeding is covered by the yellow line depicting $b$). We tested a second scale-free network with a larger base community of 120 nodes before preferential attachment of 180 additional nodes. The figures produced by the two budget-selection methods were so similar to Figs. 6 and 7 that we exclude them to avoid repetition.

Figures 6 and 7 demonstrate that for networks with heavily skewed degree distributions (e.g., scale-free networks and many real-data examples) underprediction of existing links may mislead a planner to *overspend* on seeding to achieve target cascade sizes. In such networks, heavy investments in reducing *false negative* rates may be justified. Testing this new method of choosing a slightly lower budget (still sufficient for a complete cascade in $G$) for small-world networks, our qualitative observations from Figs. 2, 3 and 4 were preserved: $\text {OAS}_{\text {mg}}$ curves simply appear to shift slightly leftwards.

**Fig. 8:**
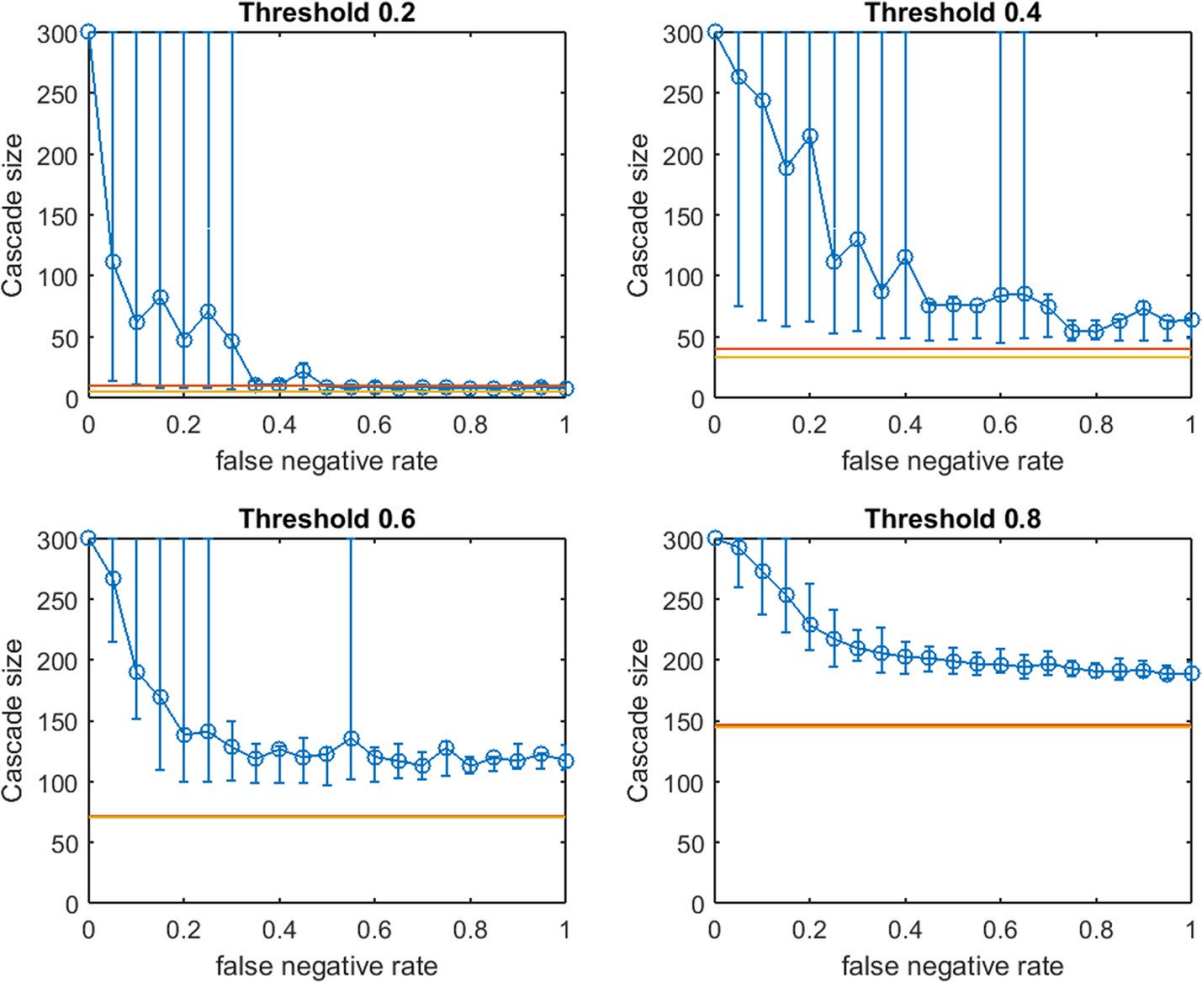
Increasing link-prediction error in a scale-free network (smaller budget, $\text {OAS}_{\text {tg}}$). The experiment from Fig. 7 is replicated with *traditional greedy*. $\text {OAS}_{\text {tg}}$ as a function of false negative rate in a scale-free network (initial society of 40 followed by preferential attachment of 260 nodes). Nodes have uniform infection thresholds 0.2, 0.4, 0.6, 0.8. Budgets are sufficient for *traditional greedy* to give a complete cascade in $G$. Budgets are (5, 33, 70, 145)

In Fig. 8 we replicate our experiment from Fig. 7 for *traditional greedy* seeding. Notably, the budgets required to give complete cascades in $G$ for *modified greedy* and *traditional greedy* are almost identical across threshold levels (Figs. 7 vs. 8). The overspending we observed by *traditional greedy* in small-world networks doe not appear to be an issue in our scale-free network examples.

Some observations about the shape of the $\text {OAS}_{\text {mg}}$ curve appear to hold for $\text {OAS}_{\text {tg}}$; however Fig. 8, exhibits a very surprising feature for higher thresholds. Namely, as $p_{\text {neg}}$ increases, $\text {OAS}_{\text {tg}}$ goes through an immediate period of steep decline—where $\text {OAS}_{\text {mg}}$ appeared robust—but then $\text {OAS}_{\text {tg}}$ appears to stabilize far above the performance of random seeding despite very-high link-prediction error. The budgets specified in Figs. 7 and 8 are very similar: while $\text {OAS}_{\text {mg}}$ has stronger performance for low $p_{\text {neg}}$, amazingly, at very high $p_{\text {neg}}$ the $\text {OAS}_{\text {tg}}$ seeding strategy consistently outperforms random seeding. Somehow, *traditional greedy* strategy is accessing useful structural insight about scale-free $G$ despite extreme departures between $E(G)$ and the observed $E(G')$. This remarkable tolerance to very noisy $G'$ is obvious at the highest thresholds ($\tau =0.6,0.8$) but also noticeable for $\tau =0.4$.

**Fig. 9:**
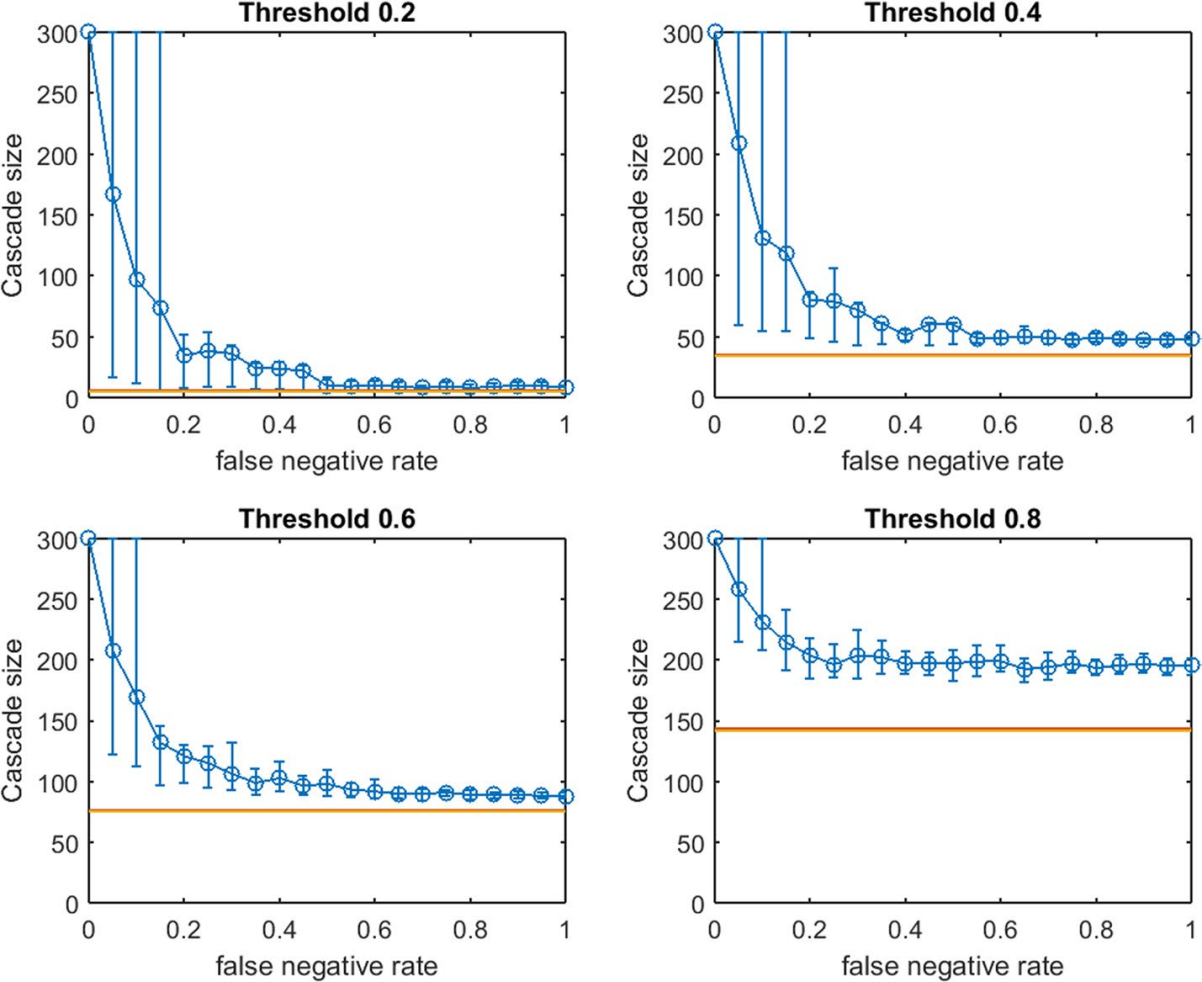
Increasing link-prediction error in a scale-free network (larger core, $\text {OAS}_{\text {tg}}$). The experiment from Fig. 8 is replicated with a scale-free network generated from a larger initial core (120 nodes, rather than 40 as in Fig. 8). $\text {OAS}_{\text {tg}}$ as a function of false negative rate in a scale-free network and all nodes have infection thresholds 0.2, 0.4, 0.6, 0.8. Budgets are (5, 34, 75, 141). Budgets are sufficient for *traditional greedy* to give a complete cascade in *G*

To test our observations from Fig. 8, in Fig. 9 we consider a second scale-free network. The initial base community has 120 nodes with average-degree 16 (binomial degree distribution). Next, 180 new nodes are added gradually to the network according to the preferential-attachment function (3). Again, while initially $\text {OAS}_{\text {tg}}$ declines steeply, at higher thresholds we note that even for extreme departures between $E(G')$ and *E*(*G*), $\text {OAS}_{\text {tg}}$ consistently outperforms random seeding attempts at the same budget (that often convert no non-seeds). The magnitude of the $\text {OAS}_{\text {tg}}$ advantage over random seeding at threshold $\tau =0.8$ for the highest $p_{\text {neg}}$ values is quite surprising.

### Real networks

#### Spanish email-exchange network

In [30], an email network of University at Rovira i Virgili was built by regarding each email address, including those of faculty, researchers, technicians, managers, administrators, and graduate students, as a node and linking two nodes if there is an email communication. We study the biggest connected component which contains 1133 nodes and 5451 edges. Since the degree distribution resembles that of a scale-free graph, to avoid over-seeding based on $G'$ (as noted in the discussion of Fig. 6), for each threshold we seed at a budget $b$ so that perfect link-information greedy seeding achieves a full cascade in] $G$ (similar to Figs. 7, 8, 9).^6^

**Fig. 10:**
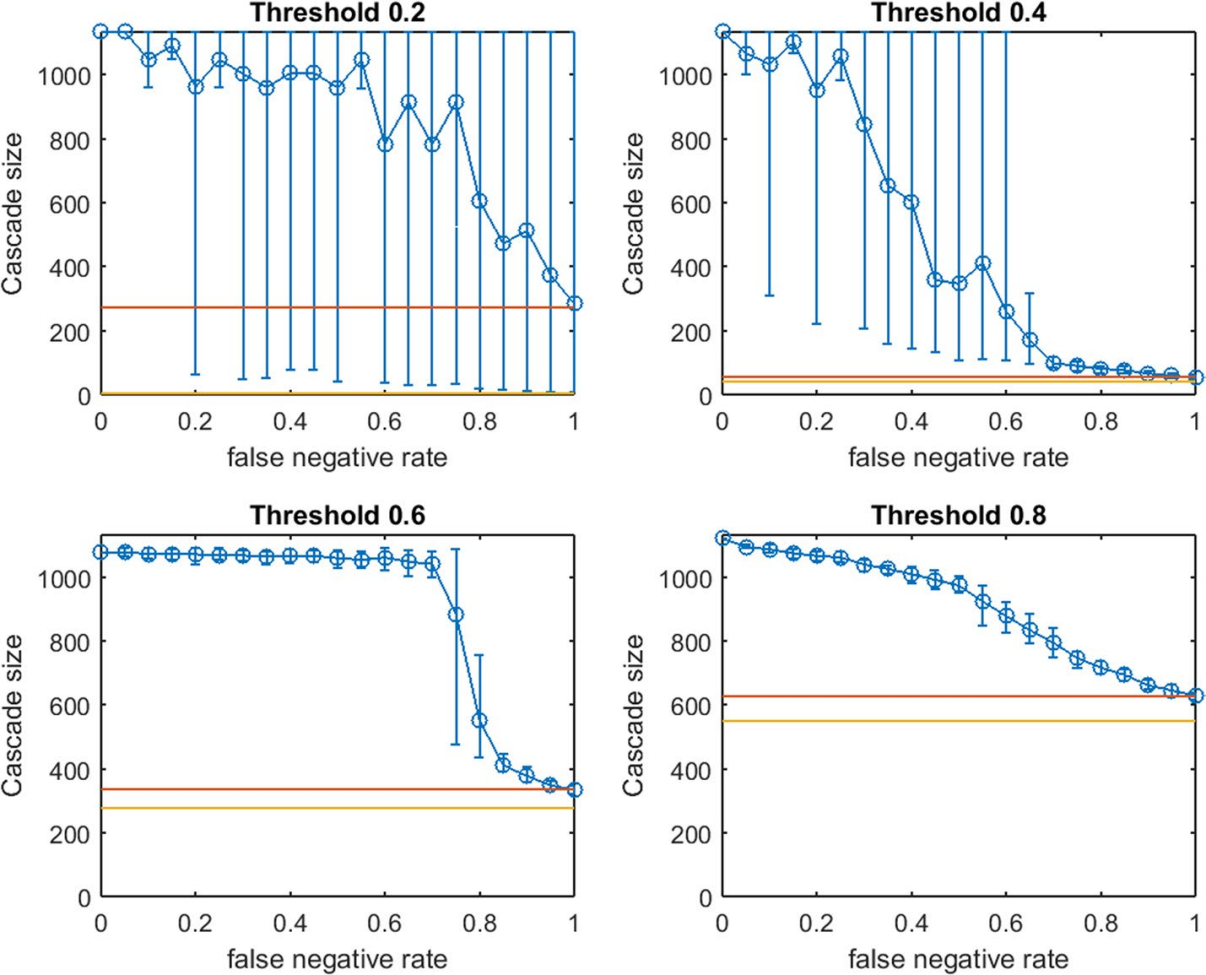
Increasing link-prediction error in an email-exchange network ($\text {OAS}_\text {mg}$ for 1133 nodes). $\text {OAS}_\text {mg}$ as a function of false negative rate in the Spanish email network and all nodes have infection thresholds 0.2, 0.4, 0.6, 0.8. Budgets are sufficient for *modified greedy* to give a complete cascade in $G$. Budgets are (4, 39, 275, 551)

**Fig. 11:**
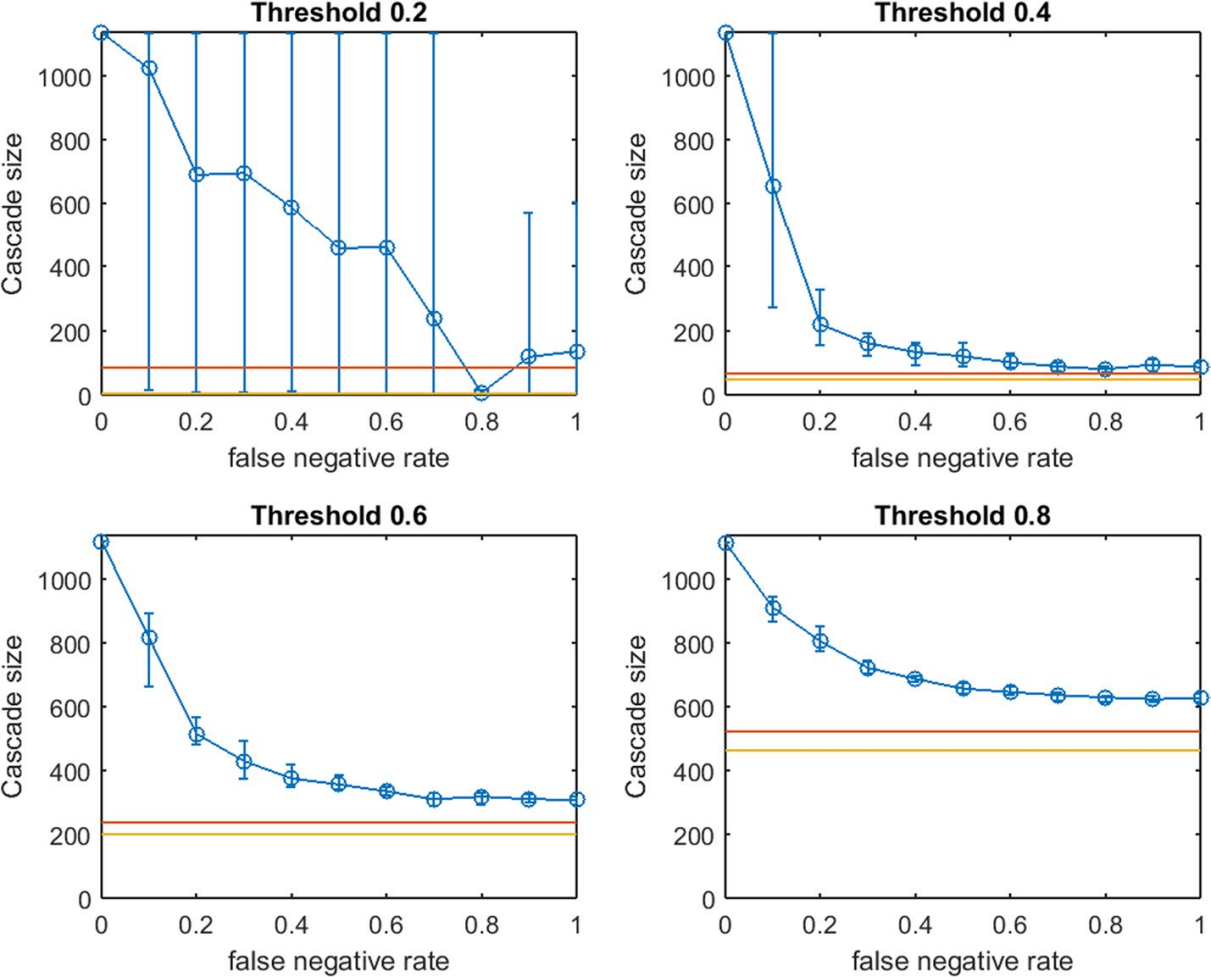
Increasing link-prediction error in an email-exchange network ($\text {OAS}_\text {tg}$ for 1133 nodes). $\text {OAS}_\text {tg}$ as a function of false negative rate in the Spanish email network and all nodes have infection thresholds 0.2, 0.4, 0.6, 0.8. Budgets are sufficient for *traditional greedy* to give a complete cascade in $G$. Budgets are (2, 48, 201, 463). Since *traditional greedy* is very slow, resolution and replication are reduced: computing cascade margins for this figure required over 30 million simulations of spread through a 1133 node graph. Five replications are conducted at each of 11 $p_{\text {neg}}$ values

As in our synthetic network tests, we observe a decline in $\text {OAS}_{\text {mg}}$ as $p_{\text {neg}}$ increases. Remarkably, except when the infection threshold is quite small, we observe that $\text {OAS}_{\text {mg}}$ reliably outperforms random seeding until $p_{\text {neg}}$ is very high. Over an initial interval, increasing $p_{\text {neg}}$ has mild impacts on $\text {OAS}_{\text {mg}}$. As $p_{\text {neg}}$ passes a critical level we again observe a steep descent to the performance level of random seeding. This is remarkably similar to what we noted in smaller synthetic networks. Threshold $\tau =0.8$ may appear to provide somewhat of an exception, but the mild erosion of performance caused immediately as $p_{\text {neg}}$ increases from 0 again is followed by an interval of slightly steeper descent (with larger variance) to match random seeding performance. We note that the distributions of cascade sizes for $\tau =0.6$ and $\tau =0.8$ are often extremely narrow.

In Fig. 11, *traditional greedy* seeding is applied to the real email network. In contrast to $\text {OAS}_{\text {mg}}$ curves from Fig. 10, $\text {OAS}_{\text {tg}}$ curves appear drop immediately as $p_{\text {neg}}$ increases from 0. Link prediction error causes immediate damage to the *traditional greedy* strategy based on $G'$. These $\text {OAS}_{\text {tg}}$ curves strongly resemble our results for $\text {OAS}_{\text {tg}}$ in smaller synthetic scale-free networks (Figs. 8, 9).

Remarkably, at higher thresholds ($\tau = 0.6, 0.8$) in Fig. 11 we again observe the remarkable stabilization of $\text {OAS}_{\text {tg}}$ performance far above the performance of random seeding (26% above random seeding for $\tau = 0.6$ and 19% above random seeding for $\tau = 0.8$). We note that no such *stabilization of* $\text {OAS}_{\text {tg}}$ *effect* was observed when $\text {OAS}_{\text {tg}}$ was applied in small-world networks (Figs. 3, 5).

Caution is warranted in making direct comparisons between Fig. 10 ($\text{OAS}_{\text {mg}}$) and Fig. 11 ($\text {OAS}_{\text {tg}}$): *modified greedy* requires a higher budget to cause a full cascade in $G$ for most thresholds: 0.2, 0.6, 0.8. In these cases, the relative lack of stability of the $\text {OAS}_{\text {tg}}$ strategy for low values of link-prediction error (e.g., $p_{\text {neg}}$ in [0.3]) may be simply due to seeding with a smaller budget. Note that for $\tau =0.4$ however, the budget for *modified greedy* (39 seeds) is much smaller than for *traditional greedy* (48 seeds), and yet $\text {OAS}_{\text {mg}}$ remains competitive with perfect link-information seeding up to approximately $p_{\text {neg}}=0.25$, and massively outperforms $\text {OAS}_{\text {tg}}$ across $p_{\text {neg}}\in [0, 0.6]$. This behavior appears to parallel stability advantages of $\text {OAS}_{\text {mg}}$ over the early $p_{\text {neg}}$ range we observed in comparing Fig. 7 ($\text {OAS}_{\text {mg}}$) and Fig. 8 ($\text {OAS}_{\text {tg}}$) for a smaller synthetic scale-free network.

#### UCI messenger-app network

 In [31], an on-line community consisting of students at the University of California, Irvine (UCI) is investigated. In the Facebook-like social network, an undirected edge is formed between two users if at least one message is sent between them. To exclude users that appear to be inactive (or barely active), we remove nodes of degree 2 or less. The resulting network contains 1281 nodes and 13,010 edges.

**Fig. 12:**
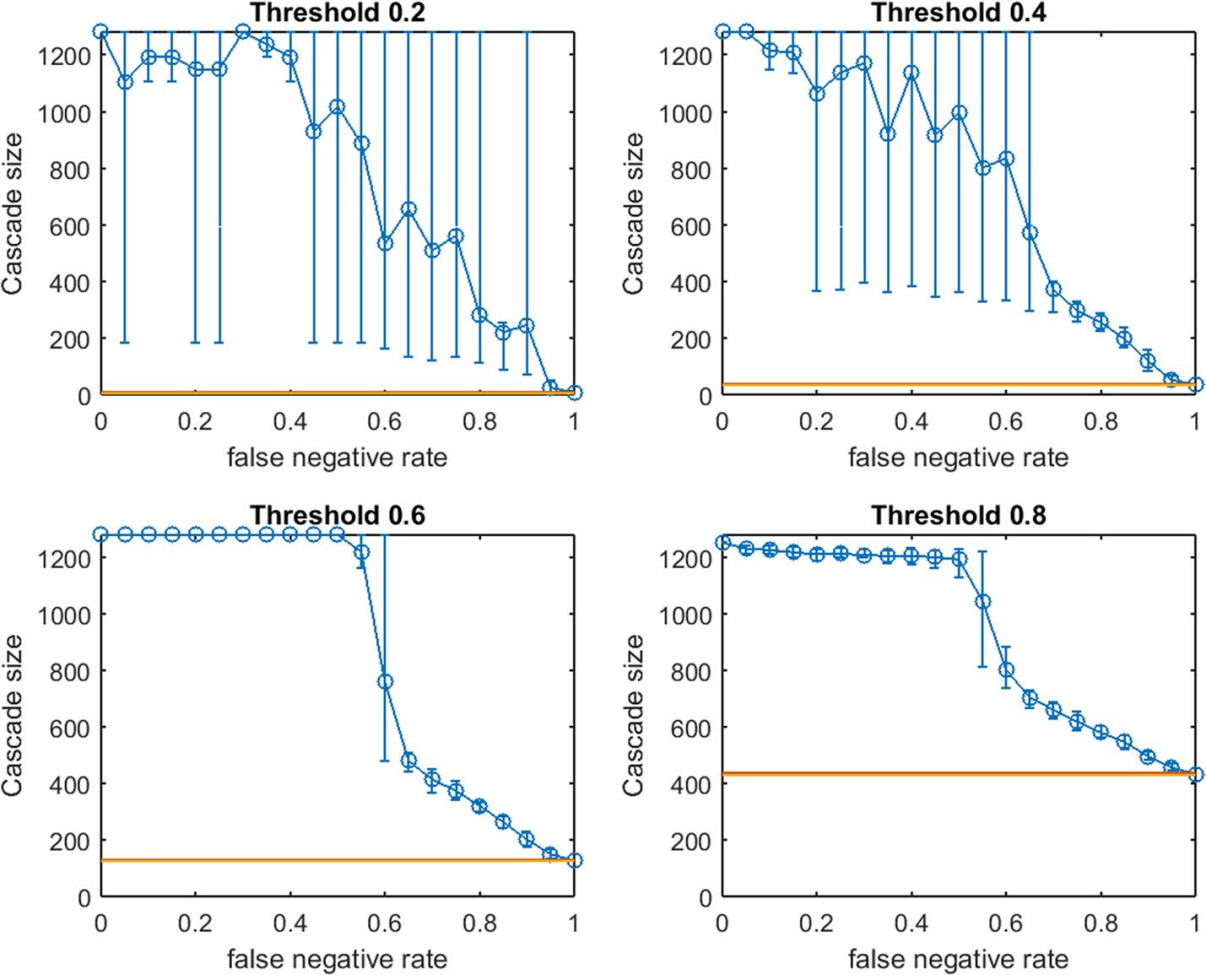
Increasing link-prediction error in a messenger-app network ($\text {OAS}_{\text {mg}}$ for 1281 nodes). $\text {OAS}_{\text {mg}}$ as a function of false negative rate in the UCI network and all nodes have infection thresholds 0.2, 0.4, 0.6, 0.8. Budgets are sufficient for *modified greedy* to give a complete cascade in $G$. Budgets are (4, 35, 125, 428)

**Fig. 13:**
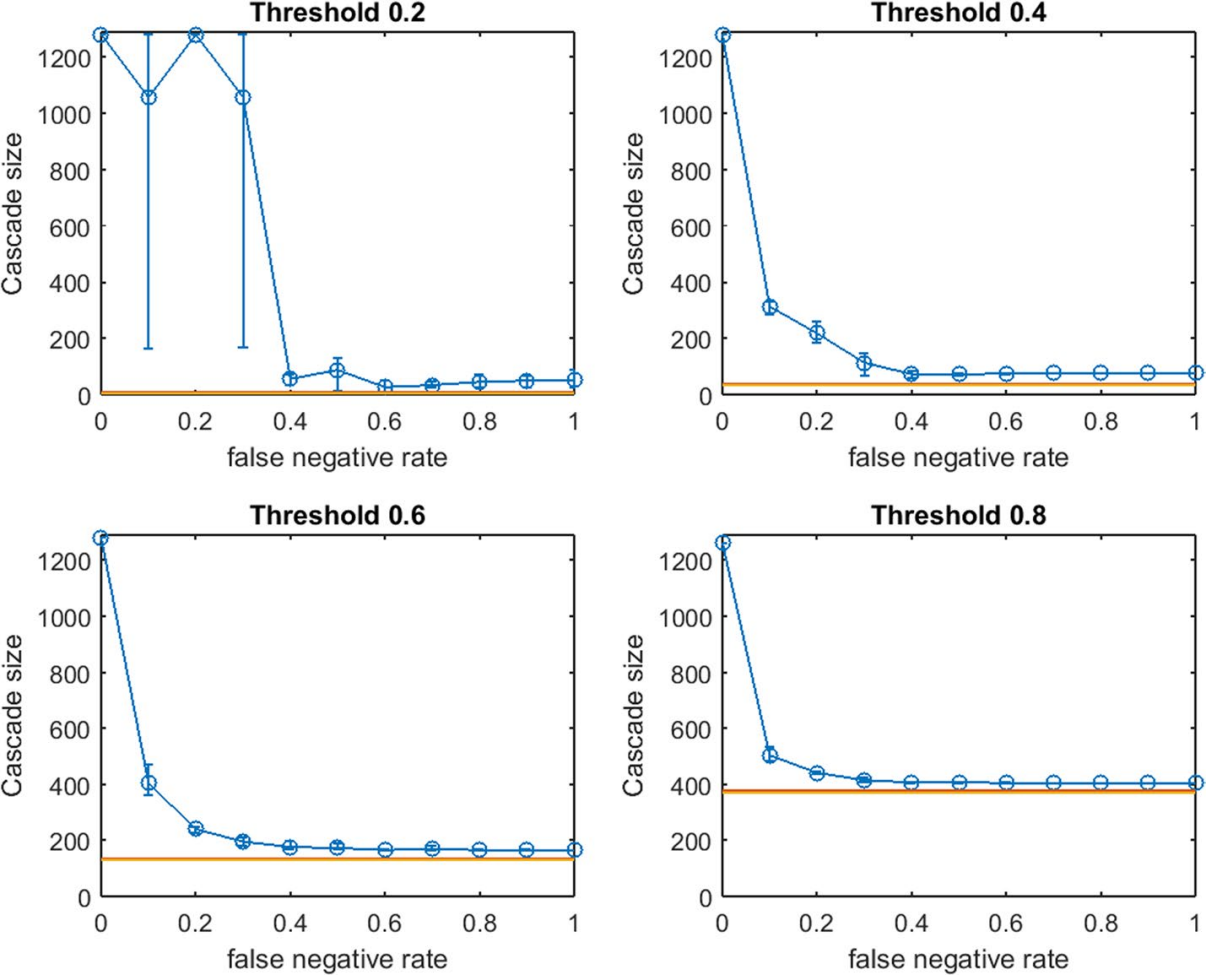
Increasing link-prediction error in a messenger-app network ($\text {OAS}_{\text {tg}}$ for 1281 nodes). $\text {OAS}_{\text {tg}}$ as a function of false negative rate in the UCI network and all nodes have infection thresholds 0.2, 0.4, 0.6, 0.8. Budgets are sufficient for *traditional greedy* to give a complete cascade in $G$. Budgets are (5, 34, 131, 371). Since *traditional greedy* is very slow, resolution and replication are reduced: computing cascade margins for this figure required over 27 million simulations of spread through a 1281 node graph. Five replications are conducted at each of eleven $p_{\text {neg}}$ values

As with the Spanish email network, we seed so that perfect-information greedy seeding gives a full cascade in $G$: how much damage is caused by imperfect link prediction? Notably, these budgets are very small for both $\text {OAS}_{\text {mg}}$ and $\text {OAS}_{\text {tg}}$: the horizontal lines that plot seeding budget $b$ (yellow) and mean random performance (red) in each of Figs. 12 and 13 almost perfectly coincide.

As in prior $\text {OAS}_{\text {mg}}$ experiments, Fig. 12 exhibits an initial period in which increasing $p_{\text {neg}}$ has mild impact, followed by a steep decline in performance. Interestingly, at lower thresholds ($\tau =0.2$, $\tau =0.4$), this decline appears more gradual (with broad distribution of performance of $V'$ in $G'$). At higher thresholds ($\tau =0.6$, $\tau =0.8$), after a long interval in which increasing $p_{\text {neg}}$ has only mild impact, we see a range where decline is very steep (similar to our observations in synthetic networks, e.g., Figs. 2, 4, 7) but this is followed by a second period of linear decline where $\text {OAS}_{\text {mg}}$ exceeds random seeding despite very-high false negative rates, $p_{\text {neg}}$. In this final period, though $\text {OAS}_{\text {mg}}$ is declining, seeding based on $G'$ is still providing reliable advantage over random seeding: distributions of cascade size are surprisingly narrow. This recalls Fig. 10 for $\text {OAS}_{\text {mg}}$ in the Spanish email network.

Figure 13 replicates the experiment from Fig. 12 but for *traditional greedy* seeding in $G'$. Though the effect is less visually obvious than in Figs. 8, 9, and 11, for $\text {OAS}_{\text {tg}}$ in the UCI Messenger-App Network we again observe some performance stabilization above random-seeding even at the highest $p_{\text {neg}}$ values: 100%+ above for $\tau =0.4$, 22% above for $\tau =0.6$, and 7% above for $\tau =0.8$.

The budgets required by *modified greedy* and *traditional greedy* seeding allow for some direct comparisons of Figs. 12 and 13. Note that $\text {OAS}_{\text {tg}}$ uses more seeds at $\tau =0.2$ and 0.6, and only one less seed at $\tau =0.4$ (34 rather than 35). Consider the corresponding subplots of Figs. 12 and 13: despite using fewer seeds, $\text {OAS}_{\text {mg}}$ performance is strong (and competitive with seeding based on perfect link information) across wide initial ranges of $p_{\text {neg}}$ values. In contrast, as $p_{\text {neg}}$ increases, $\text {OAS}_{\text {tg}}$ immediately declines steeply. This immediate erosion of $\text {OAS}_{\text {tg}}$ performance for the UCI messenger-app network is even more dramatic than we observed in the Spanish email-exchange network (Fig. 11) or in our synthetic scale-free examples (Figs. 8, 9). The estimates of $V'$ found by applying *modified greedy* seeding in $G'$ appear much more robust against link-prediction error than those found by *traditional greedy* seeding in $G'$. At the highest threshold in Fig. 12, $\text {OAS}_{\text {mg}}$ again displays almost complete stability over the range $p_{\text {neg}}\in [0,0.5]$. Unfortunately, no direct comparison is possible with Fig. 13 ($\text {OAS}_{\text {tg}}$) here: the higher $\text {OAS}_{\text {mg}}$ performance could simply be due to overspending by *modified greedy* seeding (which requires 15% more seeds at $\tau =0.8$ than *traditional greedy* seeding).

### Uniform thresholds: when does poor link prediction provide a reliable advantage?

When does the performance of a seeding strategy that is optimized-against-a-sample *reliably exceed* mean random seeding (that uses no information about *G*’s topology)? Intuitively, this should be true when $p_{\text {neg}}$ is very low, but in the figures above we observed an unexpected trend:

*As the infection threshold increases, the* $\text {OAS}_{\text {mg}}$ *strategy appears to consistently outperform the no-information random-seeding strategy even when* $p_{\text {neg}}$ *is quite high. At lower thresholds, distributions of cascade sizes under* $\text {OAS}_{\text {mg}}$ *are wide, and reliably match perfect-information greedy seeding only when* $p_{\text {neg}}$ *is very low.*

Qualitatively, it appears that at higher thresholds, *modified greedy*-optimized strategies for Uniform Threshold seeding have *increased tolerance* to link-prediction error. Our real-data examples provide the most extreme example of this observation in Figs. 10 and 12. Remarkably, despite the incredibly poor quality of the noisy network samples as $p_{\text {neg}}$ becomes large, at high thresholds this structural information is providing reliable insight in selecting high-influence seed sets.

Effectively, for high thresholds, the cascade size caused by the planner’s $\text {OAS}_{\text {mg}}$ estimate of $V'$ appears to be very stable (despite substantial differences in $E(G)$ and $E(G')$) up to a critical level of link-prediction error. Above this critical level of link error, the spatial structure of $V'$ no longer hints towards excellent seed placement in $G$. Less stability is observed at lower thresholds: as $p_{\text {neg}}$ rises, $V'$s performance in $G$ quickly decreases and becomes quite variable: the spatial structure of a good seed set in $G'$ may not indicate much about the spatial structure of a good seed set in $G$.

While a planner choosing an $\text {OAS}_{\text {tg}}$ estimate of $V'$ may struggle with some issues of *over-fitting* and *overspending* in small-world networks (Figs. 3, 5), in scale-free networks (Figs. 8, 9) and some real network datasets (in particular, Fig. 11), we observe a second style of tolerance to very high $p_{\text {neg}}$:

*As the infection threshold increases,* $\text {OAS}_{\text {tg}}$ *performance appears to stabilize reliably above the performance level of random seeding, even for the highest rates of link-prediction error. At the lowest thresholds, as link-prediction error increases,* $\text {OAS}_{\text {tg}}$ *does decline to match the random-seeding baseline. *

That is, at high thresholds in scale-free networks, it appears that even highly noisy observations of $G$ are enough for *traditional greedy* seeding to gain useful structural insight. We note that our particular model of link uncertainty (false negative vs. false positive rates) may be significant here: even for the highest $p_{\text {neg}}$, our uncertainty model is density preserving: $G'$ resembles a random graph with each edge being present with probability $p_{\text {pos}}$. Somehow this minimal signal about $G$ can be leveraged by *traditional greedy* when $G$ is scale-free, but is apparently not useful, or even damaging to the planner, when $G$ is small-world (Figs. 3, 5).

**Fig. 14:**
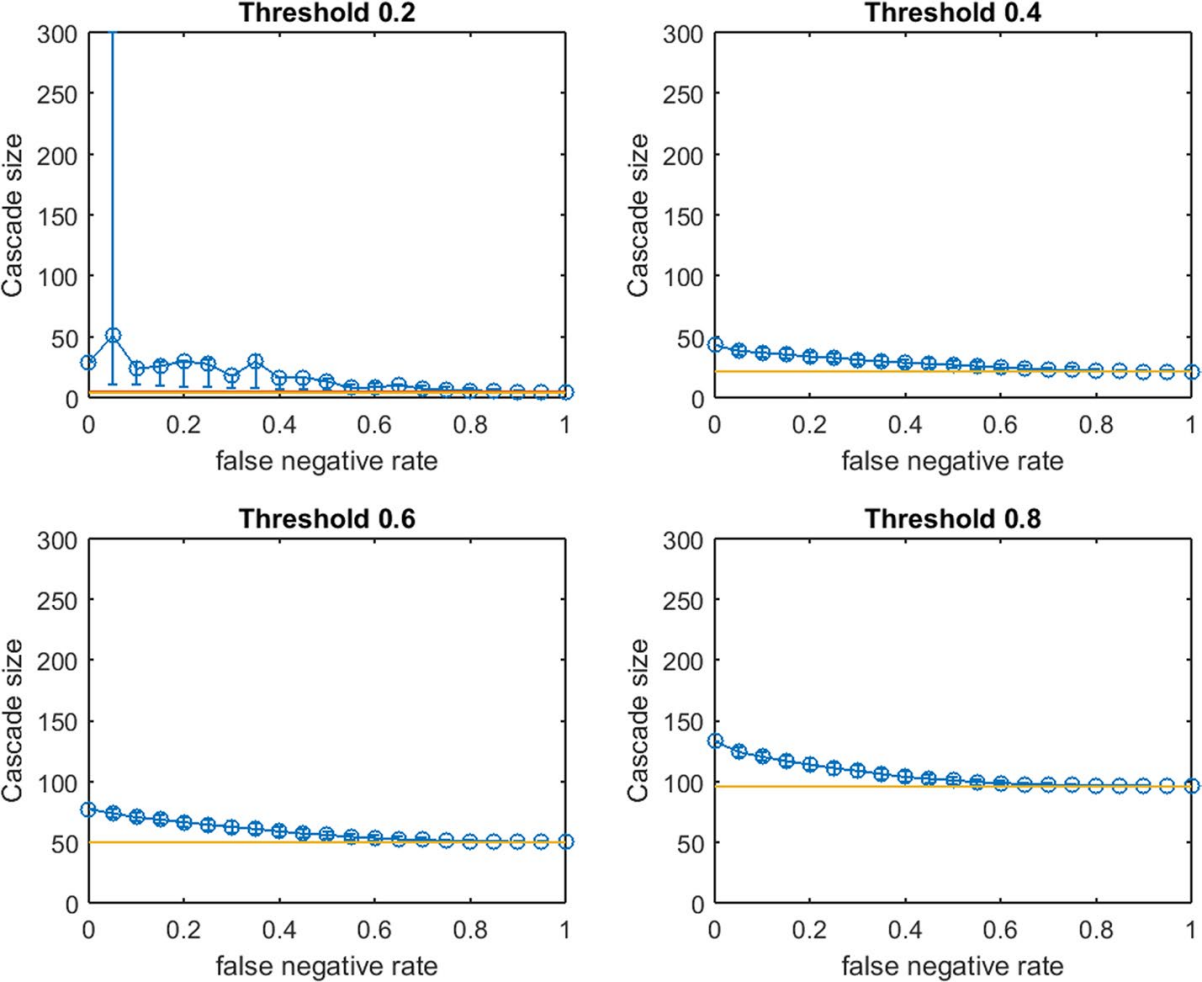
Increasing link-prediction error in a scale-free network (partial-cascade budget, $\text {OAS}_{\text {mg}}$). Replicates the experiment from Fig. 6 with half the budget. $\text {OAS}_{\text {mg}}$ as a function of false negative rate. Budget is 1/2 of what modified greedy requires to cause a full cascade in $G'$ across $p_{\text {neg}}$. Budgets are (4, 20, 47, 97)

### Budgets sufficient for only partial cascades in $G$

For each synthetic network (small-world, scale-free), we considered seeding at various fractions of the budget greedy seeding required to obtain a complete cascade in $G$. Probing several fractions in [0.4, 0.6], we repeatedly obtained figures that looked very similar to Fig. 14. To avoid repetition we include only this figure.

We note the strong contrast between the shapes of the $\text {OAS}_{\text {mg}}$ curves in Fig. 14 and those from our earlier experiments at higher budgets: these curve shapes now appear more similar to our $\text {OAS}_{\text {tg}}$ experiments (e.g., Fig. 9). Across topologies, we observe that imprecise link prediction can provide reliable $\text{OAS}$ advantage over random seeding up to moderate $p_{\text {neg}}$. As link-prediction error increases, damage to $\text {OAS}_{\text {mg}}$ performance is immediate and appears near-linear, with some diminishing-returns behavior (as in the $\tau =0.8$ panel of Fig. 12). For most fixed false negative rates $p_{\text {neg}}$, the distribution from which $\text {OAS}_{\text {mg}}$ is computed is incredibly narrow. It appears that the structural differences between $G$ and noisy sample $G'$ impact the performance of $V'$ in a very consistent way. One possible explanation for this lack of variation is that little “viral spread”—beyond infections of immediate neighbors of seeds—occurs at such low budgets.

Partial-cascade budgets for $\text {OAS}_{\text {tg}}$ appeared to give qualitatively similar results to Fig. 14, though a more systematic study across fractions in [0, 1] would be of interest.

### Optimizing-against-a-sample for the Linear Threshold Model of infection

In the previous section, a uniform known threshold was applied by each node. Next, we study threshold spread when each node selects an individual threshold uniformly in [0, 1]. This is known as the Linear Threshold Model which has been widely studied ([4] has been cited extensively, and Chen et al. provide a thorough survey [2]). We consider two partial-information cases:

- Case 1: The planner knows the random realization of threshold for every node. In this case, the planner’s uncertainty is limited to the topology of $G$, as in our prior experiments.
- Case 2: The realized node thresholds are not known to the planner. In this case, the topology of $G$ and the thresholds of the nodes are both uncertain.

Case 1 might be interpreted as a case in which some inherent properties of the individuals (e.g., demographics) give accurate predictions of their influenceability even though their network connections are unknown.

**Fig. 15:**
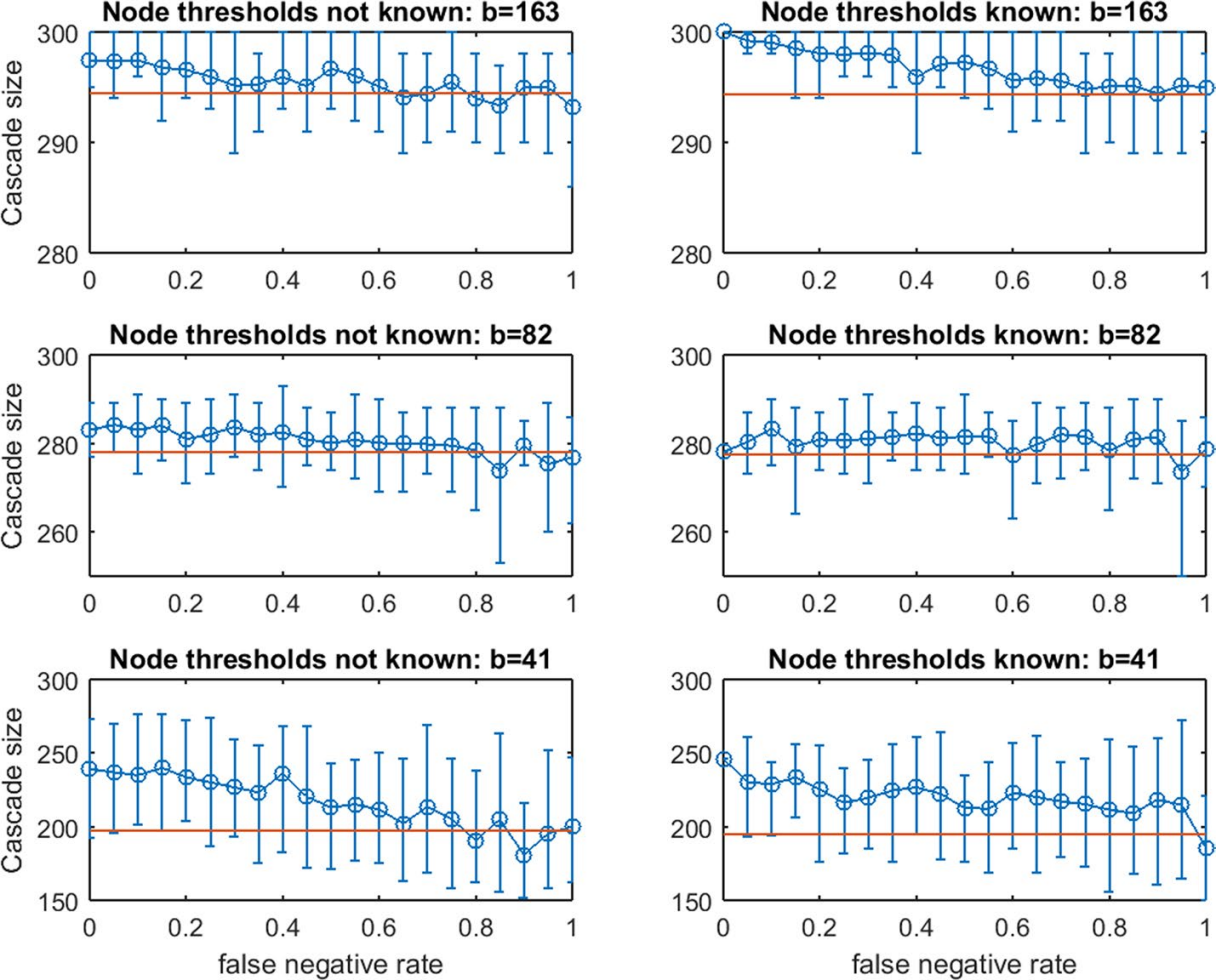
Increasing link-prediction error under linear threshold model: $\text {OAS}_{\text {mg}}$ in small-world network. Linear Threshold Model on small-world network with (*right panel*) or without (*left panel*) information on realized node thresholds. Largest budget (*top panels*) is sufficient for a full cascade under greedy seeding when realized thresholds and perfect link information are known. Half this budget and a quarter of this budget are also tested (panels labeled). Note the variable scales on the vertical axes. Mean random-seeding performance is shown in *red*

**Fig. 16:**
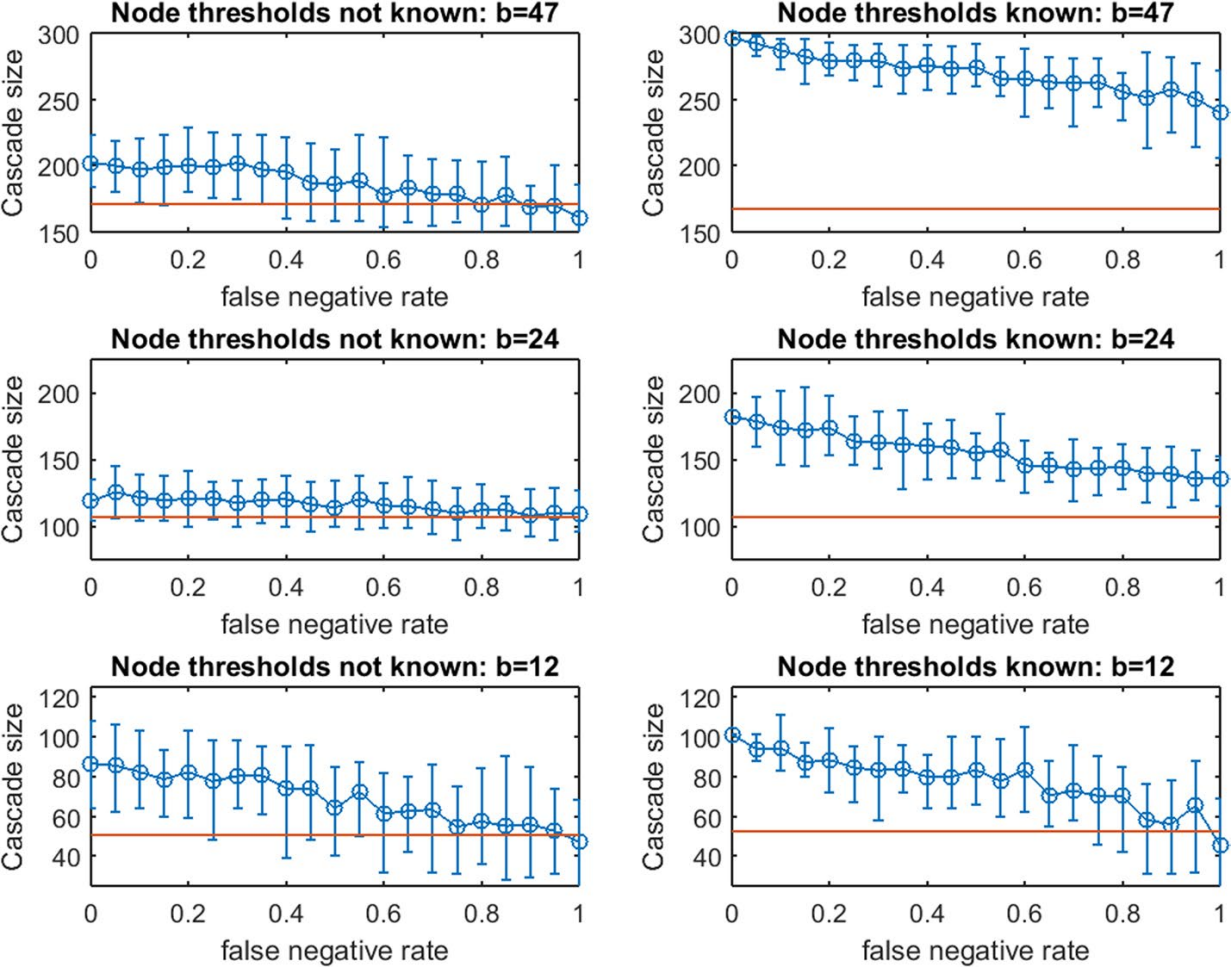
Increasing link-prediction error under linear threshold model: $\text {OAS}_{\text {tg}}$ in small-world network. Replicates Fig. 15 for *traditional greedy* seeding

**Fig. 17:**
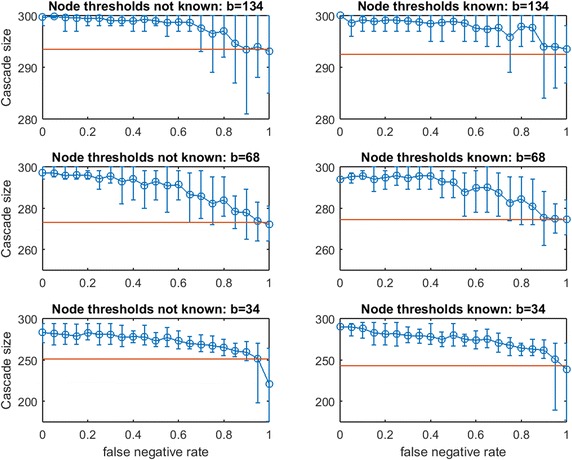
Increasing link-prediction error under linear threshold model: $\text {OAS}_{\text {mg}}$ in scale-free network. Linear threshold model on a scale-free network with (*right panel*) or without (*left panel*) information on realized node thresholds. Largest budget (*top panels*) is sufficient for a full cascade under greedy seeding when realized thresholds and perfect link information are known. Half this budget and a quarter of this budget are also tested (panels labeled). Note the variable scales on the vertical axes. Mean random-seeding performance is shown in *red*

**Fig. 18:**
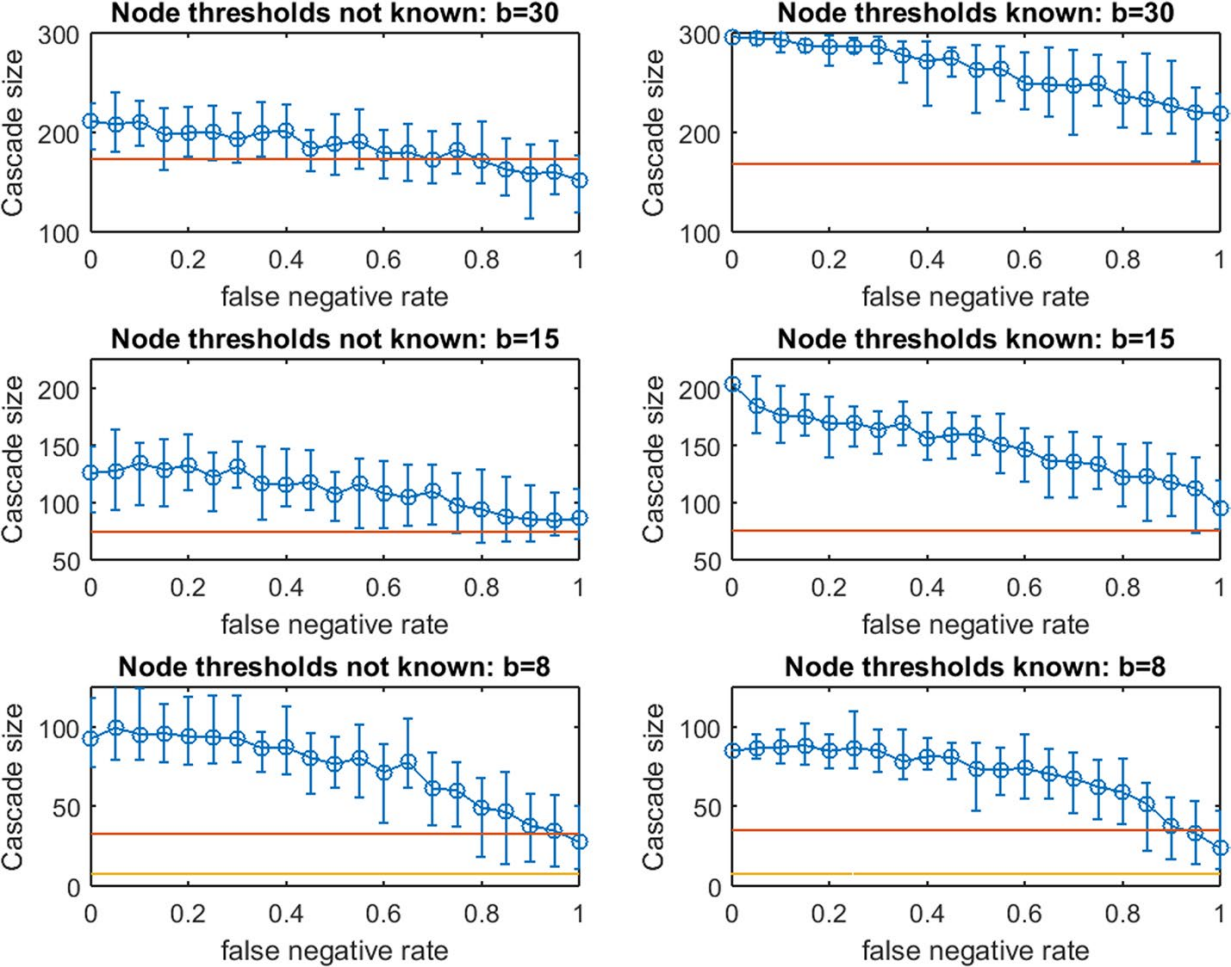
Increasing link-prediction error under linear threshold model: $\text {OAS}_{\text {tg}}$ in scale-free network. Replicates Fig. 17 for *traditional greedy* seeding

**Fig. 19:**
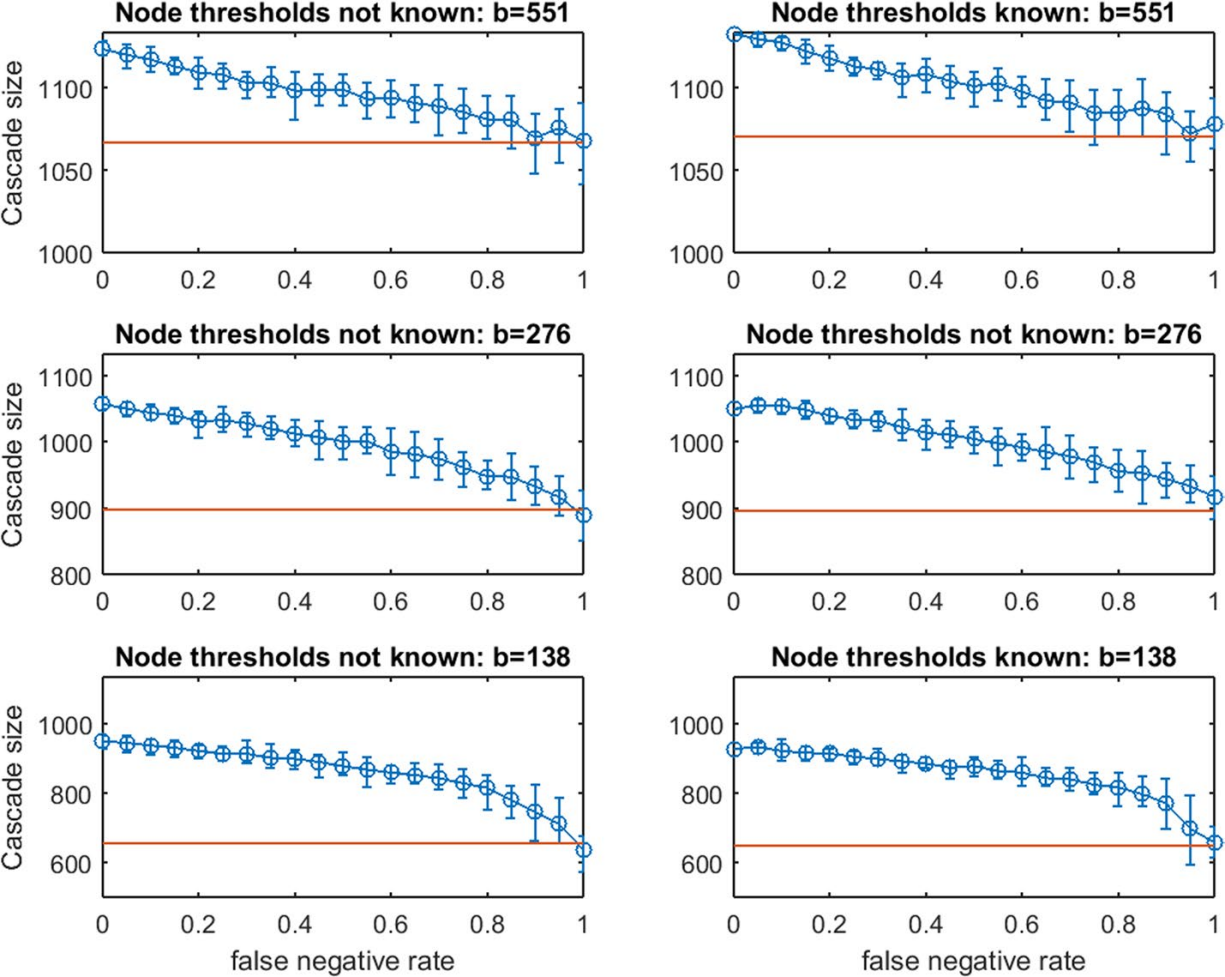
Increasing link-prediction error under linear threshold model: $\text {OAS}_{\text {mg}}$ in Spanish email network (1133 nodes). Linear threshold model on the Spanish email network with (*right panel*) or without (*left panel*) information on realized node thresholds. Largest budget (*top panels*) is sufficient for a full cascade under greedy seeding when realized thresholds and perfect link information are known. Half this budget and a quarter of this budget are also tested (panels labeled). Note the variable scales on the vertical axes. Mean random-seeding performance is shown in *red*

**Fig. 20:**
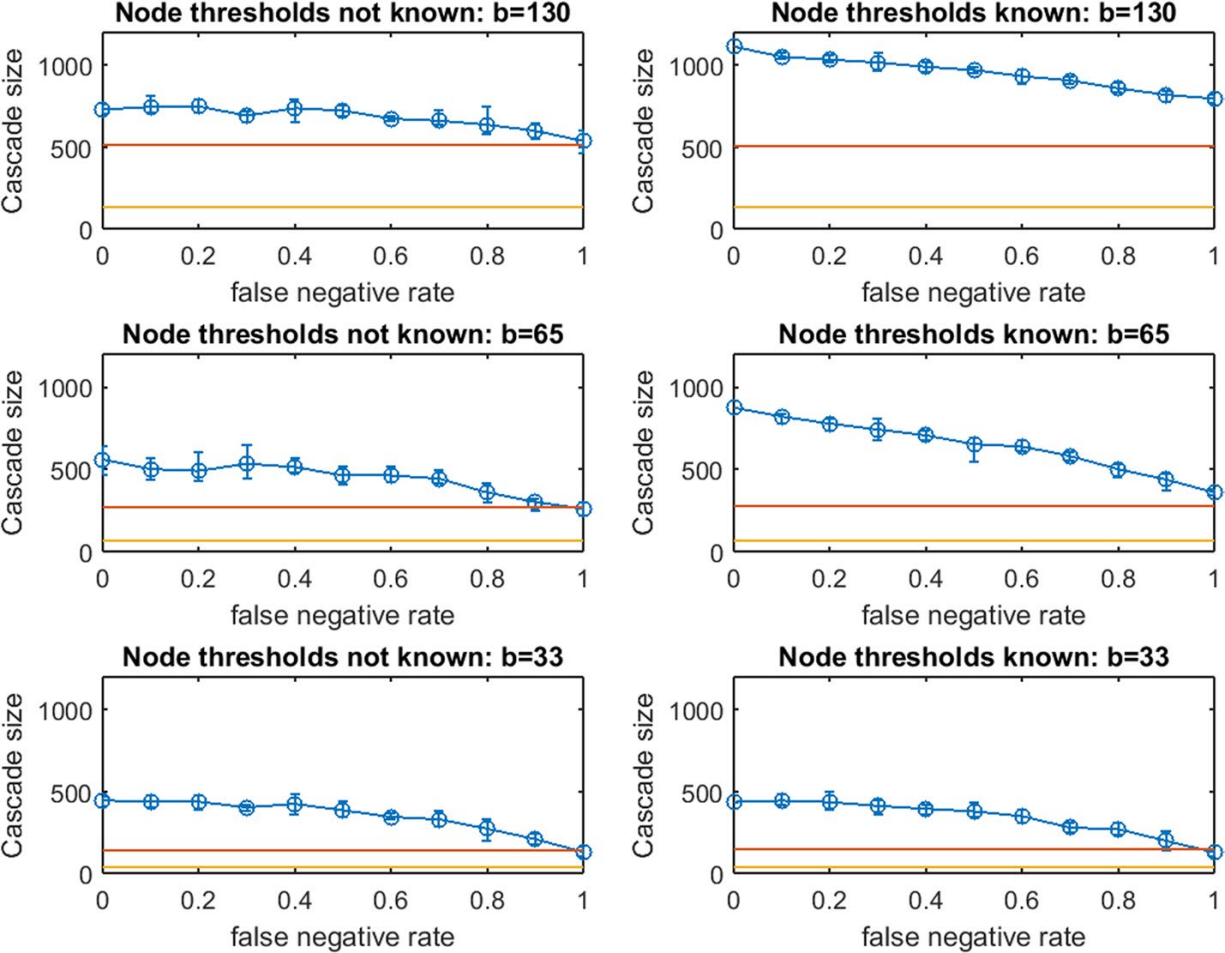
Increasing link-prediction error under linear threshold model: $\text {OAS}_{\text {tg}}$ in Spanish email network (1133 nodes). The experiment from Fig. 19 is replicated with *traditional greedy* seeding. Since *traditional greedy* is very slow, resolution and replication are reduced: this figure requires over 25 million simulations of spread through a 1133 node graph. In each subplot, five replications are conducted at each of eleven $p_{\text {neg}}$ values

**Fig. 21:**
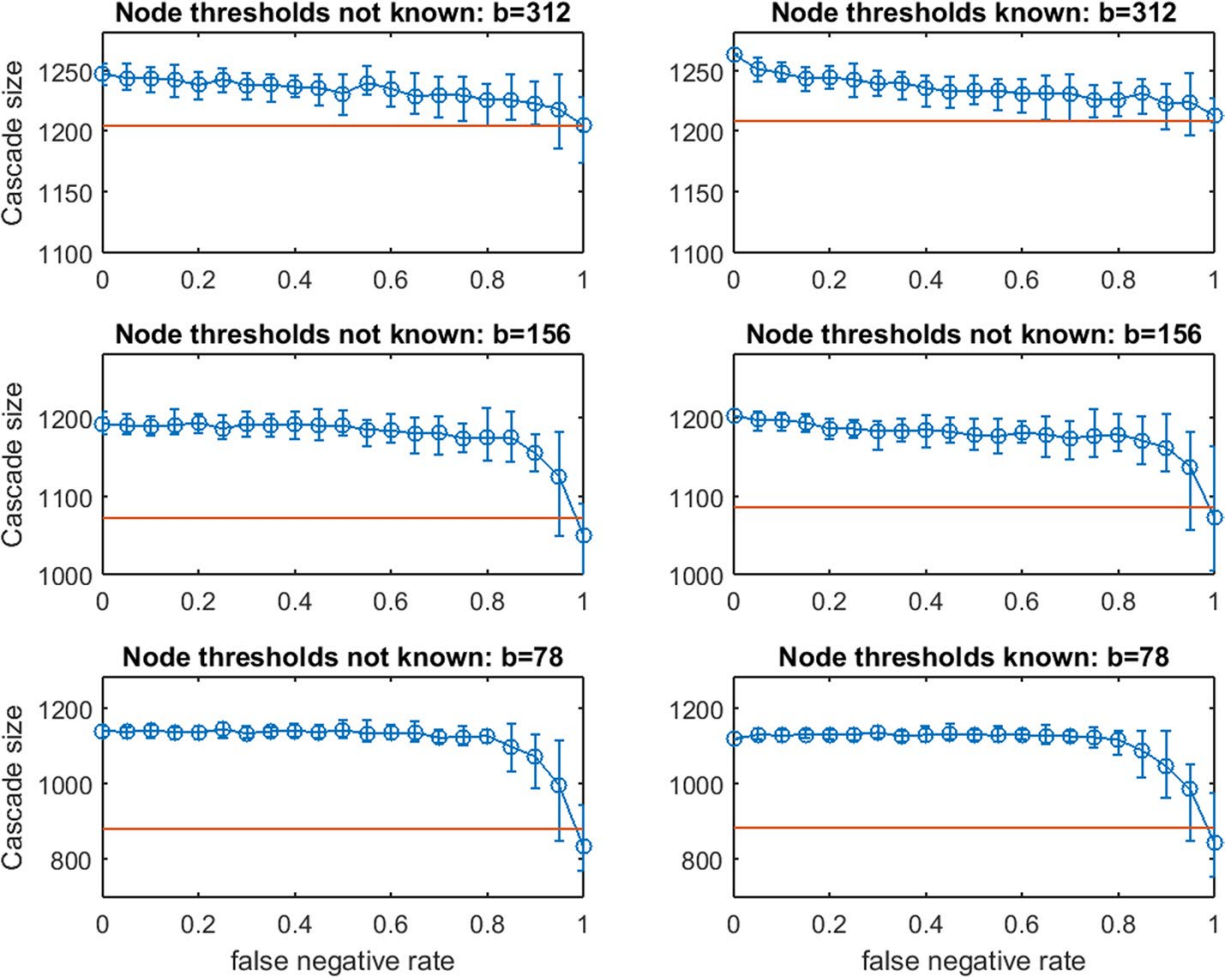
Increasing link-prediction error under linear threshold model: $\text {OAS}_{\text {mg}}$ in UCI messenger-app network (1281 nodes). Linear threshold model on the UCI messenger-app network with (*right panel*) or without (*left panel*) information on realized node thresholds. Largest budget (*top panels*) is sufficient for a full cascade under greedy seeding when realized thresholds and perfect link information are known. Half this budget and a quarter of this budget are also tested (panels labeled). Note the variable scales on the vertical axes. Mean random-seeding performance is shown in *red*

**Fig. 22:**
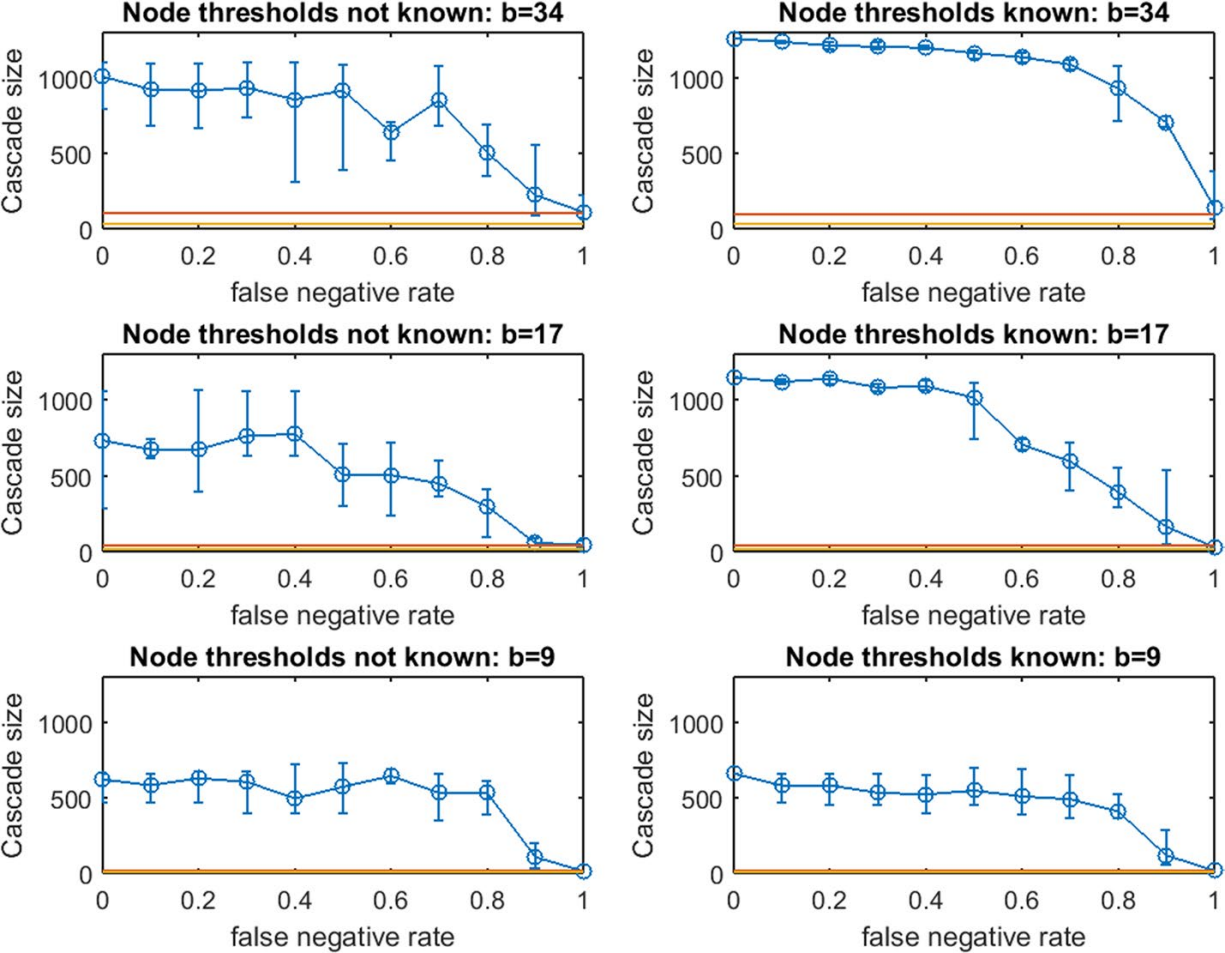
Increasing link-prediction error under linear threshold model: $\text {OAS}_{\text {tg}}$ in UCI messenger-app network (1281 nodes). The experiment from the previous figure is replicated with *traditional greedy* seeding. Since *traditional greedy* is very slow, resolution and replication are reduced: this figure requires over 8.5 million simulations of spread through a 1281 node graph. In each subplot, five replications are conducted at each of eleven $p_{\text {neg}}$ values

First we consider $\text{OAS}$ in synthetic networks for the two partial-information cases. In Figs. 15, 16, 17, 18, 19, 20, 21, and 22 we experiment at several budgets for seeding: $b$ that is sufficient in $G$ for a full cascade under greedy seeding and perfect link information, $b/2$, and $b/4$. Again, due to strong asymmetries for cascade-size distributions, we display the empirical $\text{OAS}$ estimate and the 10th–90th percentile observations of $V'$s performance in $G$ for each false negative rate.

Consider Fig. 15 of $\text {OAS}_{\text {mg}}$ in a small-world network. The *modified greedy* strategy requires a large number of seeds (163) to cause a full cascade in $G$. Given such high budgets, random seeding performs extremely well and imperfect link information appears to provide almost no advantage even when $p_{\text {neg}}$ is very low. At the lowest budget tested ($b=41$), some consistent advantage of the noisy network sample becomes visible, both when realized node thresholds are known and unknown to the planner. The only region in which Case 1 (realized thresholds are known) and Case 2 (realized thresholds are unknown) appear to differ by any meaningful additive margin is at low budget and high false negative rate. Damage to $\text {OAS}_{\text {mg}}$ performance due to increasing $p_{\text {neg}}$ appears very gradual (in strong contrast to steep $\text {OAS}_{\text {mg}}$ drops observed for the Uniform Threshold Model). We are very surprised to observe only mild departures between Case 2 (left) and Case 1 (right) for $\text {OAS}_{\text {mg}}$ panels of Fig. 15.

Figure 16 replicates the experiment from Fig. 15 for *traditional greedy* seeding. Remarkably, the budget *traditional greedy* required to cause a full cascade in $G$ is much smaller (only 47 seeds, compared with 163 seeds). This observation holds even though the set of thresholds realized in creating Fig. 16 appears “more resource intensive” than those realized in Fig. 15: the mean cascade size from 41 random seeds in Fig. 15 is roughly 200 while the mean cascade from 47 random seeds in Fig. 16 is only 165. Clearly, *traditional greedy* has a very significant advantage in seeding under the Linear Threshold Model. A planner applying $\text {OAS}_{\text {mg}}$ may significantly *overspend* when the spread process is similar to a Linear Threshold Model. The contrast between the right panels of Fig. 15 for budgets 163 and 82 also exposes this *overspending* problem: at $p_{\text {neg}}=0$, to infect less than 10 additional nodes, the *modified greed*y method requires 81 additional seeds! *Modified greedy* focuses on meeting thresholds with seed nodes only, and is blind to infections after the first time step. As the seed set is constructed, *modified greedy* adds many nodes as seeds that would already become infected through cascade. Under Uniform Threshold spread, we observed that this naive (and fast) *modified greedy* approach frequently outperformed *traditional greedy*: for Linear Threshold spread it is a substantial liability.

In Fig. 16, we observe across treatments that the advantage of $\text {OAS}_{\text {tg}}$ can be substantial and it appears to erode in a gradual linear manner as $p_{\text {neg}}$ increases. The contrast between Case 2 (left panels) and Case 1 (right panels) shows that knowledge of realized thresholds allows $\text {OAS}_{\text {tg}}$ to provide significant value even when $p_{\text {neg}}$ is very high. For example, in the top panels for budget 47, knowing node thresholds delivers a cascade-size advantage of 75–100 nodes (roughly 40–50%) across the entire $p_{\text {neg}}\in [0,1]$ range. This effect is also strong at budget $b/2= 24$, but appears to dissipate at the lowest budget $b/4=12$. Under the Linear Threshold model, *traditional greedy* seeding is able to powerfully leverage information about low- vs.-high thresholds even as knowledge about which specific pairs of nodes are adjacent becomes highly eroded.

Next, consider the analogous pair of figures for a scale-free network: Fig. 17 ($\text {OAS}_{\text {mg}}$) and 18 ($\text {OAS}_{\text {tg}}$). As in the contrast between Figs. 15 and 16 for a small-world network, we observe that *modified greedy* wastefully overspends compared to *traditional greedy*. For example, contrasting the top and bottom panels of Fig. 17: to infect roughly 15 additional nodes, *modified greedy* requires 100 additional seeds!

In Fig. 17 we observe qualitative behavior that is very consistent across budget levels: Case 1 and Case 2 again appear highly similar for $\text {OAS}_{\text {mg}}$, and $\text {OAS}_{\text {mg}}$ remains reliably above random mean performance until false negative rate is very high. Similar to the bottom panels of Fig. 15 for small-world Networks, decline in $\text {OAS}_{\text {mg}}$ appears to be remarkably shallow and gradual. Also, the distributions of cascade size are very narrow until $p_{\text {neg}}$ is high. Unfortunately, because *modified greedy* leads to such a high estimate of $b$, the margin in cascade size that can be gained from $\text {OAS}_{\text {mg}}$ seeding, while reliable, is very small in magnitude. At the lowest tested budget, $b/4=34$, this reliable $\text {OAS}_{\text {mg}}$ advantage rises to 10–15% even for quite large $p_{\text {neg}}$.

In Fig. 18, results for $\text {OAS}_{\text {tg}}$ in a scale-free network appear quite similar to our observations for $\text {OAS}_{\text {tg}}$ in a small-world network (Fig. 16). Across treatments, $\text {OAS}_{\text {tg}}$ provides reliable advantage over random seeding until $p_{\text {neg}}$ is quite large. For moderate and large budgets, knowledge of realized node thresholds allows $\text {OAS}_{\text {tg}}$ to deliver a substantial margin in cascade size (left panels vs. right panels for budgets of 30 and 15). For example, at $b=30$, across $p_{\text {neg}}\in [0,1]$, knowledge of realized node thresholds delivers an extra 35–50% margin in $\text {OAS}_{\text {tg}}$ performance. As in Fig. 16, at the lowest budget this advantage appears milder.

Next, consider Figs. 19, 20, 21, and 22 for real-data networks.

In the Spanish email network (Fig. 19 for $\text {OAS}_{\text {mg}}$ and Fig. 20 for $\text {OAS}_{\text {tg}}$), we observe strong parallels to our observations for synthetic networks. Again, *modified greedy* dramatically overspends compared with *traditional greedy* for seeding linear threshold spread. As with Figs. 15 vs. 16, and Figs. 17 vs. 18, this *overspending* in the email network is roughly a factor of 4. For the UCI messenger-app network (Figs. 21 vs. 22) we observe that *modified greedy* overspends *traditional greedy* by a factor of 9!

As with smaller synthetic networks, in real networks (Figs. 19, 21) we observe that $\text {OAS}_{\text {mg}}$ provides a reliable advantage over random seeding even when $p_{\text {neg}}$ is quite large. The magnitude of this advantage is most compelling (25%+) at the lowest budgets we test (at $b=138$ in Fig. 19, and $b=78$ in Fig. 21). In the email network (Fig. 19) erosion in $\text {OAS}_{\text {mg}}$ is remarkably mild as $p_{\text {neg}}$ increases, and this effect is exaggerated in the messenger-app network (Fig. 21) where $\text {OAS}_{\text {mg}}$ performance appears completely stable until the highest $p_{\text {neg}}$ values. We suspect that this stability in Fig. 21—and the remarkably small variance of cascade sizes—may indicate that until link-error is extreme, $\text {OAS}_{\text {mg}}$ is able to identify a seed set that infects a stable set of large clusters in the UCI messenger-app network. At the highest $p_{\text {neg}}$, the $\text {OAS}_{\text {mg}}$ strategy starts to fail to reliably infect some of these communities.

For $\text {OAS}_{\text {tg}}$ in real networks, we see strong connections to our observations in small synthetic networks. While $\text {OAS}_{\text {mg}}$ shows negligible differences between Case 1 (known node thresholds) and Case 2 (unknown node thresholds), for $\text {OAS}_{\text {tg}}$, knowledge of node thresholds provides a substantial additional performance margin (compare left panels to right panels in Figs. 20 and 22). Just as in small synthetic networks, this margin for Case 1 is substantial at $b$ and $b/2$, and appears to dissipate at the lowest budget tested ($b/4$) for both real network datasets.

Even without knowledge of realized thresholds, $\text {OAS}_{\text {tg}}$ provides a large advantage over random seeding. In the email network (Fig. 20), at $p_{\text {neg}}=0.4$ this advantage grows from roughly 40% at the highest budget ($b=130$) to 300%+ at the lowest budget ($b=33$). In particular, across budget levels, $\text {OAS}_{\text {tg}}$ cascade sizes are competitive with the perfect link-information case until $p_{\text {neg}}$ is quite large. Even at very large $p_{\text {neg}}$, erosion of $\text {OAS}_{\text {tg}}$ performance is gradual.

In the UCI messenger-app network (Fig. 22), the budget required by *traditional greedy* is very small: $\text {OAS}_{\text {tg}}$ massively outperforms random seeding at every budget level we test until the highest $p_{\text {neg}}$ values. As in the Email network, $\text {OAS}_{\text {tg}}$ remains competitive with the perfect link-information seeding until surprisingly large $p_{\text {neg}}$. As we speculated for $\text {OAS}_{\text {mg}}$ in Fig. 21, the stability of cascade sizes across a wide range of increasing $p_{\text {neg}}$ (e.g., for $p_{\text {neg}}\in [0,0.7]$ in the bottom right panel of Fig. 20) may be due to $\text {OAS}_{\text {tg}}$ infecting some stable set of large clusters as long as $G'$ is not *too different* from $G$. Eventually, $G'$ departs too strongly from $G'$, $\text {OAS}_{\text {tg}}$ no longer reliably infects these clusters, and performance declines somewhat quickly.

### Discussion of contrasts

The Uniform Threshold Model and the Linear Threshold Model lead to very different messages about the value of accurate link prediction in optimizing seeding.

- *Uniform threshold model* At budgets sufficient to cause full cascades, $\text{OAS}$ appears to behave very differently at low and high thresholds.
  For $\text {OAS}_{\text {mg}}$, Figs. 2, 4, 7, 10, and 12 show that as threshold increases, there is an increasing range of error in link prediction that can be *tolerated* without $\text {OAS}_{\text {mg}}$ losing much efficacy. In this range, investments in improving link prediction provide minimal advantage to the planner and may be wasteful. Under *modified greedy* seeding, the transition from noisy $G'$ informing near-optimal seeding strategies in $G$ to being almost useless in reasoning about $G$ is sudden: $\text {OAS}_{\text {mg}}$ declines steeply at a *critical level of link-prediction error*. For spreading low-threshold phenomenon, very accurate link prediction is essential for seeding based on $G'$ to reliably deliver high performance in $G$ (even when $\text {OAS}_{\text {mg}}$ is high, the distribution of $V'$s performance may be widely variable). For spreading high-threshold phenomenon, greedy seeding based on quite-noisy link prediction can still reliably identify high-performing seed sets. For a planner facing high-threshold spread, investments in improving link prediction can be highly non-linear: pushing $p_{\text {neg}}$ below the *critical level* can massively boost cascade sizes planned based on $G'$. Changes in $p_{\text {neg}}$ that do not bridge this *critical level* have only mild impacts on the cascade sizes obtained from $\text {OAS}_{\text {mg}}$ seeding. In strong contrast, at lower budgets that allow only partial cascades (where infection fails to “go viral”), damage caused by imperfect link prediction appears to exhibit “diminishing returns” for all topologies across a wide range of threshold levels.
  Under *traditional greedy* seeding, or $\text {OAS}_{\text {tg}}$, results in small-world networks were similar to $\text {OAS}_{\text {mg}}$ though possible issues with overspending are observed in Fig. 5. In scale-free networks, $\text {OAS}_{\text {tg}}$ exhibited a surprising different style of tolerance for very-high link-prediction error: after a period of steep $\text {OAS}_{\text {tg}}$ performance decline, for higher node thresholds, we observed that $\text {OAS}_{\text {tg}}$ performance stabilized significantly above the random seeding baseline (Figs. 8, 9). This observation appeared to anticipate a similar effect in our real network datasets (Fig. 11, and to a milder extent, Fig. 13). Thus, if link-prediction error is already low, investments to reduce error further could provide significant margins in cascade size, but at high link-prediction error these investments would be wasted (even though highly noisy views of $G$ allow the planner to significantly outperform random seeding).
- *Linear threshold model* While $\text{OAS}$ based on *modified greedy* frequently outperformed *traditional greedy* for Uniform Threshold spread, for Linear Threshold spread, $\text{OAS}$ based on *traditional greedy* exhibits compelling advantages. First, *modified greedy* wastefully overspends compared with *traditional greedy* for all synthetic and real networks we study. A planner attempting to estimate a strategic budget based on $G'$ seems to be much better served by an $\text {OAS}_{\text {tg}}$ approach. Second, $\text {OAS}_{\text {tg}}$ is able to leverage information about realized node thresholds to achieve major gains in cascade size (while $\text {OAS}_{\text {mg}}$ appears unable to extract value from this additional source of information).
  For scale-free-like networks (synthetic and real), we did find that until departures between $G'$ and $G$ are severe, $\text {OAS}_{\text {mg}}$ can reliably yield some advantage (Figs. 17, 19, 21). The magnitude of this $\text {OAS}_{\text {mg}}$ advantage was somewhat limited as random seeding at the same budget levels was also quite successful. This appeared to be consistent over a range of budgets. We observe two behaviors. In the synthetic scale-free network and Spanish email network, damage caused by link-prediction error appears very gradual: investments in reducing $p_{\text {neg}}$ have relatively small uniform impact regardless of the current value of $p_{\text {neg}}$. Though the UCI-Messenger-app degree distribution also resembles a scale-free degree distribution, at lower budgets the shape of the $\text {OAS}_{\text {mg}}$ curve exhibits stability over a broad range of increasing link-prediction error rates, followed by a sudden steep decline. Qualitatively this is reminiscent of our observations for the Uniform Threshold Model: a *modified greedy*-chosen seed set based on $G'$ is somehow extremely stable under high link-prediction error for this real network example. We hypothesize that this difference arises from some mid-level structure of the UCI messenger-app network. Interestingly, $\text {OAS}_{\text {tg}}$ in the UCI messenger-app network (Fig. 22) might lead to a similar hypothesis. For all other topologies (Figs. 16, 18, 20), $\text {OAS}_{\text {tg}}$ performance exhibits gradual shallow decline as $p_{\text {neg}}$ increases. In contrast, Fig. 22 seems to exhibit initial flatter regions (where $\text {OAS}_{\text {tg}}$ remains highly competitive with perfect link-information greedy seeding), followed by steeper regions where $\text {OAS}_{\text {tg}}$ erodes to the random-seeding baseline.

Finally, we note that for uniform thresholds, the shape of $\text {OAS}_{\text {mg}}$ curves appears to depend strongly on the budget for seeding, while $\text {OAS}_{\text {tg}}$ curves appeared more consistent in shape at various partial-cascade budgets. This was observed repeatedly in widely differing topologies. In contrast, under linear thresholds, the shape of the $\text{OAS}$ curves for a fixed network and fixed greed-seeding algorithm appeared more consistent regardless of budget.

## Conclusion

Intuitively, as link-prediction error rises, the value of a noisy network observation should decline. For both greedy-seeding methods we study, when seeding a viral-marketing campaign that spreads at low uniform thresholds, investing in highly accurate link prediction appears essential. In contrast, if the uniform threshold for spread is higher, then even marginal link-prediction capability can provide value.

Surprisingly, we observe that under *modified greedy* seeding even poor link prediction delivers substantial gains in planning complete cascades for Uniform Threshold spread (both in terms of exceeding the performance of random seed selection, and in terms of matching the performance achievable with highly accurate link prediction). It appears that at higher thresholds, the spatial form of high-performing seed sets is more robust against variation in the precise network topology. This pattern, visible in our synthetic test networks, appears very strong in the real-network datasets we test.

For *traditional greedy* seeding in scale-free networks (including two larger real network datasets), we observe a different style of spatial robustness of seeding strategies. It appears that at higher uniform thresholds, while initial link uncertainty is highly damaging to performance, the value of a very noisy network observation *stabilizes*, leading to cascade sizes significantly above the performance of random seeding even for very-high link-prediction error.

When instead spread is based on node-specific thresholds that are distributed uniformly in [0, 1] (the Linear Threshold Model), we observe that even very noisy network observations provide substantial value. For most topologies (small-world, scale-free, and a real email network) link-prediction error appears to cause gradual linear damage to cascade sizes. Still, in one large real network example (the UCI messenger-app network), we do observe remarkable stability of cascade sizes until quite high link-prediction error, followed by a steeper regions of cascade-size decline.

Our study suggests that the value of accurate link prediction in network seeding depends closely on the spread mechanism to be seeded: even the apparently similar variants of threshold spread studied in this paper point toward different rules of thumb. We summarize these observations qualitatively in the following table.

**Question: invest in reducing link-prediction error?**

**Table:** 

Spread mechanism	Low link-prediction error ($p_{\text {neg}}$)	High link-prediction error ($p_{\text {neg}}$)
High uniform Infection threshold	$\text {OAS}_{\text {mg}}$ competitive with perfect-info	$\text {OAS}_{\text {mg}}$ near random seeding
	$\text {OAS}_{\text {tg}}$ declines steeply, overspends	Scale-fr: $\text {OAS}_{\text {tg}}$ beats random seeding
	Small $b$: error reduction is mild gain	Small $b$: error reduction is no gain
	Large *b*: error reduction is low/no gain	Large $b$: large gain opportunity
Low uniform Infection threshold	$\text{OAS}$ high, but wide distribution	$\text{OAS}$ near random seeding
	Small $b$: mild gain opportunity	Error reduction is low gain
	Large $b$: modest/large gain opportunity	
Linear threshold Uniform [0, 1]	Recommendation: use $\text {OAS}_{\text {tg}}$ (requires much smaller budgets than $\text {OAS}_{\text {mg}}$)	
	At a range of budgets: $\text {OAS}_{\text {tg}}$ reliably beats random seeding until highest $p_{\text {neg}}$	
	Link-error reduction only mild/modest gain: instead invest to learn node thresholds	
	Observed real-data exception for UCI Messenger-App Network: at a range of budgets, large gain opportunity for link-error reduction at high $p_{\text {neg}}$	

In a practical marketing context, early stage investigation of the success of spread at different levels of peer exposure (and variability across individuals) may critically inform the optimal level of investment a company should make in improving link-prediction error and what seeding algorithms should be applied in observed or estimated networks. In considering strategic levels of investment in link prediction, the planner should also consider their budget, $b$. The size of cascades being planned appears to strongly impact the value of good link prediction under the Uniform Threshold Model: in key parameter ranges, large premiums in cascade size may be gained by investing in improved link prediction. In other ranges, $\text{OAS}$ performance appears quite insensitive to improvements in link prediction: such investments would be wasted.

In contrast, under the Linear Threshold Model, improvements in link prediction appear to usually provide mild-or even low-linear gains in cascade size (regardless of the seeding budget). Since $\text{OAS}$ with moderate link-prediction error reliably locates high-performance seed sets, if the planner suspects that a Linear Threshold Model describes spread well, investments in highly accurate link prediction may not be justified. Instead, if the planner is able to implement *traditional greedy* seeding (or some close approximation)^7^, investments in learning more about node-specific thresholds (perhaps tied to demographic factors, or observable via past campaigns) might provide higher returns in cascade size.

We note some limitations of our study and comment on possible future work. Our main finding deals with how the value of a noisy network sample varies as a function of infection threshold. This inquiry requires the ability to vary infection threshold somewhat smoothly. In networks where a majority of nodes have very low degree (so that thresholds like 0.4 and 0.6 are functionally identical), our results will necessarily be eroded. Future work could also investigate the value of seeding strategies that are based on noisy network observations that overestimate the density of the network (many “friends” may not be trusted for product recommendations, etc), or that distort the relative degrees of nodes (e.g., some demographics are easier to overpredict links for than others). Also, the authors would be interested to see further studies that consider a finer-scale investigation of budgets that achieve large, but incomplete, cascades.

Our computational study of $\text{OAS}$ has considered $\text {OAS}_{\text {mg}}$ and $\text {OAS}_{\text {tg}}$. These are only two of the methods a planner might use to estimate $V'$ from noisy sample $G'$. In general, these estimates of $V'$ may be quite different from truly optimal seed sets in $G'$ (except when $V'$ is optimal for budget $b$ in the sense that $V'$ gives a full cascade in $G'$, and no other seed set of size $b$ could give a larger cascade in $G'$—as in Figs. 2, 3, 4, 5 and 6). As we have discussed, significant differences in $\text{OAS}$ behavior emerged as a result of the seeding algorithm applied in $G'$, and some differences appeared to suggest rich interactions between the seeding method and the network topology (e.g., Figs. 7 vs. Fig. 8). From a theoretical perspective, it is not clear that any particular algorithm-dependent measurement will accurately reflect on true $\text{OAS}$ performance, nor that, given the complexity issues involved in accurately computing $V'$, a fully accurate computational study of $\text{OAS}$ is possible except in very small networks. Nevertheless, we believe that $\text{OAS}$ is a useful concept that motivates a variety of interesting directions. Here, limiting the number of seeding methods studied allowed us to explore several variations on threshold, spread model, and network topology. Fixing a spread model and topology and experimenting with a range of methods for selecting $V'$ in the noisy network would be of great interest. In particular, our experiments reflect on the stability of two certain styles of greedily chosen $V'$ under link error, but there is no obvious reason that all methods of selecting “near-optimal” seed sets in $G'$ should have similar stability properties. It would be of great practical interest if some algorithms consistently produced $V'$ with better stability against link-prediction error, particularly if $\text{OAS}$ performance was the mean of a very narrow distribution (so that attempts to near-optimally seed based on $G'$ rarely failed).

## Figures and Tables

**Fig. 23:**
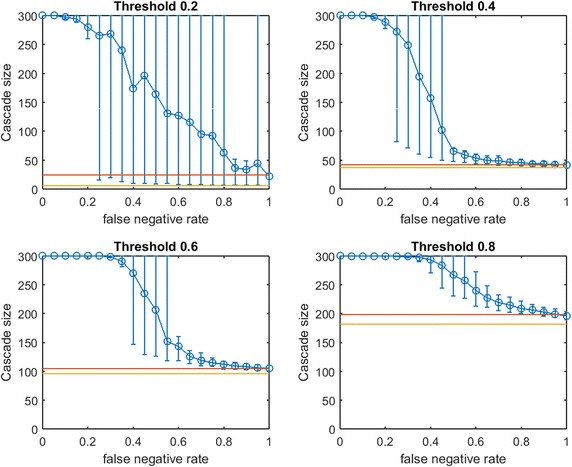
Increasing Link Prediction Error in a small-world Network (Higher Rewiring *p*, $\text {OAS}_{\text {mg}}$). $\text {OAS}_{\text {mg}}$ as a function of false negative rate in a small-world network (rewiring probability of 0.6, mean community size of 10, standard deviation 5). Nodes have uniform infection thresholds of 0.2 (*upper left*), 0.4 (*upper right*), 0.6 (*lower left*), 0.8 (*lower right*). Larger link-prediction error is modeled by larger false negative rate.* Horizontal lines* plot seeding budget $b$ and mean performance of a randomly selected seed set. *Modified greedy* allocation of $b$ seeds with perfect link prediction causes a complete cascade in $G'$ (300 nodes). Budgets are (6, 37, 96, 182)

## Appendix

Here we include an additional Fig. 23.

## Footnotes

1 This will be described in detail in “Methods”.

2 Experiments to explore the effect of link-prediction error that significantly over- or underpredicts network density would also be of interest and would be useful to describe prediction challenges around inactive or infrequently active social connections.

3 Though the planner applies a naive greedy seed-selection method in $G'$, since $V'$ causes a complete cascade in $G'$, $V'$ is by definition optimal in $G'$ among seed sets of cardinality $|V'|$.

4 A similar figure for $\text {OAS}_{\text {mg}}$ in a small-world network with higher rewiring probability appears in “Appendix.”

5 In Fig. 1, such budget levels correspond to budgets to the right of where random performance intersects optimized seeding in $G$.

6 In fact, since large real networks often contain some small almost-isolated components, we set $b$ to achieve a $98\%$+-rather than $100\%$- cascade in $G$.

7 This may not be possible for large networks.
